# CABNas-nir: A near-infrared classification for urban pipe network sludge on the fusion algorithm of NAS framework and active learning

**DOI:** 10.1371/journal.pone.0339347

**Published:** 2025-12-19

**Authors:** Yuxi Yang, Li Fu, Qingjun Wei, Yuanfa Feng, Ling Zhu, Yan Dai, Wu Xiao, Ting Fan, Xiu Jin

**Affiliations:** 1 POWERCHINA Eco-Environmental Group Co., Ltd., Shenzhen, China; 2 POWERCHINA Zhongnan Engineering Corporation Limited, Changsha, China; 3 School of Information and Artificial Intelligence, Anhui Agricultural University, Hefei, China; 4 College of Resources and Environment, Anhui Agricultural University, Hefei, China; National Research and Innovation Agency, INDONESIA

## Abstract

Pipe network sludge is a complex pollutant aggregate deposited during long-term operation of urban sewage pipelines, and a key target for pollution control in environmental monitoring systems. Accurate source classification is critical for treatment optimization, pollution tracing, and resource recovery. Traditional methods have drawbacks like long processing time and low efficiency. Near-infrared spectroscopy (NIR) offers a new approach but faces spectral redundancy, limited samples, and biased features. This paper proposes CABNas-nir, a deep neural network under the neural architecture search (NAS) framework, integrating competitive adaptive reweighted sampling (CARS), baseline drift augmentation, and active learning (AL). It selects key spectral features via CARS to remove redundancy, uses baseline drift to generate augmented samples for small-sample issues, employs AL with K-means to select high-value samples, and constructs an optimal convolutional neural network(CNN)+long short-term memory(LSTM) model via NAS. Experiments show 92.86% accuracy, 14.29% higher than support vector machine (SVM,78.57%) and 35.72% higher than that of extreme gradient boosting (XGBoost,57.14%). SHapley Additive exPlanations (SHAP) analysis shows high-contribution spectra in 1400–1700 nm, with 1600–1700 nm significant. This algorithm significantly enhances the robustness of identifying the sources of pipe network sludge, laying a research foundation for the rapid and accurate identification of pipe network sludge.

## 1. Introduction

Pipe network sludge is a complex aggregate of pollutants gradually deposited during the long-term operation of urban sewage pipeline systems. Its composition is not only highly diverse but also exhibits significant variability. In addition to containing a large amount of basic nutrients such as organic matter, nitrogen, and phosphorus, it often entraps heavy metal ions, pathogenic microorganisms, microplastics, and various industrial residual chemicals. The combination ratio of these components varies significantly with factors such as the area where the pipeline is located, sewage sources, material, and service life. Pipe network sludge from different regions differs in the content of basic nutrients like organic matter, nitrogen, and phosphorus, and may also contain signature substances [1]. Its organic matter composition is mostly related to the decomposition characteristics of domestic waste [2]. Therefore, accurate classification and identification of the source regions of pipe network sludge are of great significance. From the perspective of treatment optimization, sludge from different sources has different degradation characteristics and treatment requirements due to compositional differences [3,4]. After classification, treatment processes can be optimized targetedly, improving treatment efficiency and significantly reducing treatment costs. In terms of environmental pollution source tracing, if sludge leakage pollution occurs, classification can quickly lock in the source region, providing solid evidence for formulating pollution control measures and identifying responsibilities, thereby reducing losses caused by pollution diffusion. In terms of resource recovery and utilization, sludge from different sources has different resource potential (for example, sludge from some regions, due to the adaptability of organic matter composition to plant needs, is suitable for use as base fertilizer in landscaping). After classification, it can accurately match utilization paths, enhancing resource value [5].

However, traditional methods for classifying sludge regions mostly rely on manual sampling followed by laboratory component analysis, combined with empirical judgment of the source region. These methods have problems such as long-time consumption, strong subjectivity, and low efficiency, making it difficult to meet the needs of rapid and accurate classification. The emergence of NIR provides a new idea to solve this dilemma. This technology can quickly obtain characteristic information of internal chemical components by analyzing the absorption characteristics of sludge samples to near-infrared light. The entire detection process does not require destructive treatment of samples, realizing true non-destructive analysis. At the same time, the detection cycle is shortened from several days with traditional methods to a few minutes, greatly improving classification efficiency. Moreover, the analysis process is less affected by human factors, and the results are more objective and stable [6].These fast, non-destructive, and green technical characteristics exactly meet the dual requirements of timeliness and accuracy for sewer pipe sludge region classification, laying a technical foundation for efficient sludge classification.

Nevertheless, when using NIR for pipe network sludge region classification, the obtained spectral data contains a large amount of wavelength point information, including both characteristic information that can distinguish sludge from different regions, and a large amount of irrelevant information and noise. Irrelevant information and noise come from various sources, such as instrument fluctuations, differences in sludge physical properties, and external environmental interference [7]. If raw spectral data is directly used for classification modeling, it will increase model complexity, extend training time, and may reduce classification accuracy and stability due to the influence of irrelevant information and noise, leading to a decline in the model’s ability to distinguish sludge from different regions. Therefore, feature extraction is a key link in pipe network sludge region classification. Through feature extraction algorithms, key features that can effectively distinguish sludge from different regions can be extracted from complex spectral data. Effective feature extraction can improve the classification efficiency and accuracy of the model, enabling the model to distinguish sludge from different source regions more accurately [8].

Although the key features screened by feature extraction algorithms have removed redundant information, limited by the actual conditions of pipe network sludge sample collection (such as uneven distribution of sampling points and scarcity of samples under extreme working conditions), there are often problems such as limited sample size and obvious bias in feature distribution. This will cause the classification model to easily fall into overfitting during training, with insufficient recognition ability for unseen samples (such as sludge collected in different seasons) and limited generalization performance. Therefore, expanding and optimizing the extracted key features through data augmentation technology has become an important link to improve model stability. The design of the data augmentation scheme needs to be closely aligned with the characteristics of the research object [9]. Specifically, in the spectral analysis of pipe network sludge, considering the particularity of its spectral characteristics—spectral signals are easily affected by factors such as environmental temperature and humidity, pipeline material adsorption effects, and detection instrument precision fluctuations, showing regular variations such as baseline drift and slight peak shifts—this study adopts baseline drift as the core data augmentation method [10].This method simulates the slow drift of spectral baselines that may occur in actual detection, generating a series of augmented samples with slight differences but conforming to real physical variation laws while retaining the core information of key features (such as characteristic wavelength positions and peak intensity ratios) screened by the CARS algorithm. This augmentation method not only expands the total number of samples but, more importantly, enriches the distribution pattern of the feature space by reproducing spectral variation patterns in actual scenarios, enabling the model to learn more comprehensive variation laws of sludge spectral features during training and providing a more representative data basis for subsequent AL to screen high-value samples.

Although data augmentation can expand the sample size, the quality of generated samples varies. Some samples may fail to effectively reflect the characteristic differences of sludge from different regions, and may even interfere with the model’s learning of regional features, reducing classification accuracy [11].Therefore, in this study, an AL method is used to screen the most valuable samples from the augmented samples, i.e., those that can best help the model distinguish sludge from different regions. Through specific sampling strategies, it selects samples with the highest uncertainty for the model for training, allowing the model to focus more on learning key differences between regions. It not only reduces the interference of low-quality samples, enabling the model to quickly grasp the unique features of sludge from different regions and improve classification efficiency but also complements data augmentation, expanding the sample size while ensuring sample quality and enhancing the performance of the classification model.

Comprehensively addressing the above problems and challenges, this paper proposes a fusion algorithm (CABNas-nir) of CARS algorithm, baseline drift, and AL under the NAS framework. Our main contributions are as follows: (1) The CABNas-nir method framework is proposed, which effectively identifies the source regions of sewer pipe sludge by integrating the CARS algorithm, baseline drift augmentation, and AL; (2) A series of data experiments are carried out to verify the CABNas-nir method. Through in-depth analysis of a large amount of experimental data, it is found that the sludge recognition ability under this method is significantly improved, with an accuracy rate of 92.86%; (3) The baseline drift augmentation method is used to perform baseline drift data augmentation on the key features selected by CARS, and combined with the AL K-means clustering sampling strategy, the most valuable samples are selected from the augmented samples, effectively improving the robustness of identifying sludge source regions in complex sewer environments, and providing a promotable technical paradigm for small-sample, high-variability spectral classification tasks.

## 2. Related work

In this section, we comprehensively review the relevant literature on the research status of pipe network sludge, NIR, data augmentation, AL, and NAS, which are closely related to the focus of our research.

### 2.1. Research status of pipe network sludge

Pipe network sludge refers to solid waste formed by the natural deposition of impurities due to low water flow velocity in urban drainage pipelines (including sewage pipes, rainwater pipes, and combined drainage networks). Its sources involve multiple input pathways such as surface runoff, industrial wastewater carriers, and pipeline corrosion. Unlike sewage sludge generated in wastewater treatment plants, the formation process of pipe network sludge runs through the entire life cycle of pipeline transportation, featuring characteristics of high water content, high inorganic matter content, and complex pollutant components (including heavy metals, microplastics, pathogenic microorganisms, etc.). Owing to its hidden deposition location, uneven distribution, and tight coupling with the pipeline structure, traditional sampling methods have to rely on manual underground operations, which are associated with high risks, high costs, and low efficiency. This constitutes the core bottleneck that has caused research on pipe network sludge to lag behind that on sewage sludge for a long time.

However, if pipe network sludge is not cleaned for a long time and accumulates in pipelines, it will cause multiple problems. On one hand, it leads to the deterioration of pipeline functionality: sedimentation reduces the effective cross-sectional area of pipelines, increases the hydraulic friction coefficient, impairs drainage capacity, accelerates pipeline corrosion, and shortens service life. On the other hand, it results in the spread of environmental pollution: anaerobic fermentation produces gases such as hydrogen sulfide and methane, which corrode pipelines and escape to endanger residents’ health; during rainstorms, heavy metals and microplastics are washed into water bodies, polluting the environment. Meanwhile, it causes the accumulation of microbial risks: pathogenic microorganisms multiply in large numbers in anaerobic environments, far exceeding the limits of discharge standards.

Therefore, how to realize the resource utilization of pipe network sludge has become a current research hotspot, and its potential is reflected in its multi-component characteristics. For example, inorganic components can be processed to extract fine sand through separation technologies for use in building materials. Pipe network sludge from residential areas with high organic matter content, rich in easily degradable components such as humus and fatty acids, is suitable for conversion into biogas via anaerobic digestion processes to achieve energy recovery. Moreover, digestion residues, after composting, can be used as garden soil, and their nutrients such as nitrogen, phosphorus, and potassium can effectively improve vegetation survival rates. With the development of machine learning (ML) technology, data-driven methods have provided new ideas for pipe network sludge research [12,13]. Chen et al. [14] employed classification-based PMF and ML to establish an accurate identification method for sludge pollution sources. Specifically, due to the differences in sludge pollution characteristics between urban and rural wastewater treatment plants (T1 and T2) and the variations in sewage pipeline distribution, by classifying sludge samples and correlating pollutant concentrations with potential pollution sources, they accurately identified the electroplating industry as the primary source of sludge pollution in the study area, and found that zinc concentration was significantly correlated with the distance from electroplating factories. Samkhaniani et al. [15] utilized a ML approach (LightGBM model combined with feature selection and uncertainty analysis) to establish a reliable and efficient predictive framework for biogas production in wastewater treatment plants. Specifically, since biogas production is a complex nonlinear process affected by multiple factors such as sludge characteristics and operational practices, traditional models have limitations. By integrating collected actual data with the model, conducting exploratory data analysis, feature importance evaluation, and uncertainty quantification, they developed a predictive model that can explain 82% of the variance in the test data and identified key factors influencing biogas production.

### 2.2. Near-infrared spectrum

In recent years, NIR has been widely promoted as a rapid and non-destructive detection technology, and it has also been extensively applied in the detection of sludge characteristics. Lu et al. [16] developed an efficient, accurate, and cost-effective BMP prediction model for various types of sludge using near-infrared spectroscopy. Specifically, each type of organic solid has its unique spectrum and BMP value. By correlating the BMP value of each sample with its NIR, a BMP prediction model based on NIR can be developed. Gibouin et al. [17] determined the rheological parameters of 36 sludge samples, conducted near-infrared spectral measurements, and employed the partial least squares algorithm to establish a calibration model, evaluating the potential of near-infrared spectroscopy in predicting sludge rheological parameters. Soriano-Disla et al. [18] explored the feasibility of using NIR as a simple, low-cost, and rapid method to predict the stability parameters of sewage sludge and the resulting compost. In sewage sludge, the prediction of ash content was successful, and this work demonstrated the potential of NIR in predicting certain parameters related to the stability of sewage sludge and its derived compost. Lu et al. [19] rapidly and accurately obtained moisture content information through NIR, addressing the problem of spectral line distortion caused by moisture differences when detecting wet soil using LIBS. Combined with a calibration model, the detection accuracy of total potassium was significantly improved, ultimately realizing on-site rapid quantitative detection of nutrients in wet soil. Fu et al. [20] took the contaminated farmland soil around the Mojiang gold mine in Yunnan as the research object, using Vis-NIR as the core data source, combined with data preprocessing, feature extraction, and modeling methods, and further integrating DWT and deep learning (DL) models to significantly improve prediction accuracy, realizing the quantitative estimation of soil chromium (Cr) content, thus providing an efficient technical scheme for soil pollution monitoring. Peng et al. [21] combined NIR with multi-preprocessing and ensemble learning, breaking through the limitations of traditional detection methods, realizing rapid and accurate online monitoring of COD values in industrial wastewater, and providing key technical support for water quality management, process optimization, and ecological protection. In the above studies, NIR technology was used, and its characteristics of rapidity, efficiency, simplicity, and non-destructiveness make it more practical than traditional physical and chemical analysis methods.

### 2.3. Data augmentation

Data augmentation techniques, which simulate physical disturbances in real scenarios or introduce algorithm-generated virtual samples, have become a core means to address issues of sample scarcity and feature bias in spectral data analysis. In recent years, research in this field has shown a significant trend of multi-technology integration and scenario-adaptive optimization.

In terms of technical approaches for spectral data augmentation, researchers have proposed diverse solutions. Zhang et al. [22] developed an advanced DL model for predicting water pollutants using spectral data augmentation techniques. To address the issue that DL models struggle to fully extract and learn data features when sample data is limited, thereby affecting prediction performance, the encoder module based on the Informer architecture enhanced various frequency-domain features, and distillation technology was used to further improve feature quality and their correlation with labels. Subsequently, an improved generative adversarial network was introduced to expand the limited dataset, solving the problem of small sample sizes and improving the overall quality of the dataset. Uladzislau Blazhko et al. [23] proposed a spectral data augmentation technique called Extended Multiplicative Signal Augmentation (EMSA). Its core is to generate artificial samples by simulating common physical distortions in infrared spectra (such as scattering and instrument effects). Based on the Extended Multiplicative Signal Correction (EMSC) framework, EMSA augments datasets by simulating distortions caused by physical factors in spectral measurements (e.g., baseline shifts, scattering, instrument noise), enabling powerful models like CNNs to establish more accurate boundaries between classes. To tackle the class imbalance problem, Zhang et al. [24] (noted that obtaining large amounts of spectral data is difficult due to limitations such as the number of clinical cancer cases and spectral acquisition costs, which ultimately restricts the performance optimization and improvement of diagnostic models. Facing these challenges, they adopted different data augmentation strategies to obtain more usable training data. In addition to common augmentation methods in vibrational spectroscopy—such as adding random noise, random changes in offset, multiplication, and slope, as well as Synthetic Minority Over-sampling Technique (SMOTE)—two generative adversarial networks with different architectures were selected for comparison: one based on artificial neural networks (ANN) and the other on convolutional neural networks (CNN).At the technical integration level, Kamini G. Panchbhai et al. [25] used spectral augmentation and advanced ML to detect amylose content in rice samples. To solve the class imbalance problem in the original spectral dataset, they employed SMOTE as a spectral augmentation method, generating synthetic specimens for minority classes by interpolating between existing specimens. In synergy with spectral preprocessing and feature selection, it ultimately improved the performance across all classes.

In the above studies, spectral augmentation techniques generate a large number of “virtual samples” by reasonably transforming the spectra of existing samples, making up for the deficiency of real samples. Thus, in scenarios with limited samples and complex interference, they enhance the generalization ability, noise resistance, and feature learning efficiency of the models.

### 2.4. Active learning

AL selects information-rich samples from data for labeling using different sampling strategies, thereby improving model performance under the same labeling budget. Multiple studies on AL have been conducted in the field of NIR. Nikolaos L. Tsakiridis et al. [26] pointed out that reliable and cost-effective solutions are required to monitor the status of soil ecosystems, determine the spatiotemporal scope of applied pressures, and mitigate the impacts of climate change and land degradation. They combined AL with NIR spectroscopy to enhance the prediction accuracy of soil properties (e.g., organic carbon, clay content) in unlabeled areas. Using a pool-based AL (pool-based AL) framework combined with semi-supervised learning (Laplacian SVR), they iteratively selected the most valuable samples from unlabeled NIR spectral data for labeling to optimize model performance in new regions. He et al. [27] detected the characteristics of blended gasoline via NIR spectroscopy and noted that the estimation and generalization performance of NIR models largely depend on the quality and quantity of training samples. Thus, they proposed an active training sample selection strategy. By screening representative samples and dynamically adapting to changes, they actively selected optimal training samples from historical data using algorithms such as weighted error functions and realized online model updating through double weighting and hypothesis testing, which reduced labeling costs while improving the accuracy and adaptability of NIR models in industrial processes. Julius Krause et al. [28] estimated multiple quality and maturity parameters of agricultural products from NIR spectra using standard chemometric models. They improved the performance of chemometric calibration models while reducing redundant reference analyses by optimizing sample selection. Huang et al. [29] proposed a model named Safer-AS, which is safer and more effective compared to similar models, given the challenge of acquiring sufficient labeled spectral samples to meet modeling requirements. The above studies all focus on the application of AL in the NIR spectroscopy field. By optimizing sample selection strategies, they improved model performance while controlling labeling costs, providing more efficient and economical solutions for NIR spectral analysis in different scenarios.

### 2.5. Neural architecture search

In addition to the quality of sample data, model efficiency is also closely related to its parameter settings. Therefore, accurately configuring the optimal parameter combination of the model is of particular significance [30,31]. Hence, this study employs NAS to construct the network structure and determine its parameters. NAS introduces a differentiable architecture search method, which accelerates the search process by optimizing the hyperparameters of the network structure using gradient descent, enabling faster and more accurate task completion. NAS has been widely applied in numerous scenarios. Chen et al. [32] deeply integrated NAS with the requirements of embedded devices based on the idea of “customized search space + optimized search unit + model lightweighting”: they improved classification accuracy by leveraging the automated architecture design capability of NAS, and reduced resource consumption through improvements such as quantization and feature fusion, ultimately achieving efficient recognition of Diptera insects on embedded devices. Yang et al. [33] realized the automated design of multi-task spectral analysis networks via genetic algorithm-driven NAS. The search space focuses on feature extraction and task interaction, adapting to the high-dimensional characteristics of spectral data; the genetic algorithm efficiently screens optimal module combinations through evolutionary operations, avoiding the limitations of manual design. Experiments on ginseng and wheat flour datasets showed that this method can significantly improve multi-task prediction accuracy, verifying the practicality of NAS in spectral analysis. Ye et al. [34] applied NAS technology to field pest detection in a targeted manner through customized search spaces, evolutionary mechanisms, and hybrid search algorithms, realizing automatic optimization of the architecture and solving problems such as poor adaptability of manually designed architectures and difficulty in small target detection.

## 3. Materials and methods

### 3.1. Sample collection and data analysis

This study selected Shunde District, Foshan City, Guangdong Province (23°02′N, 113°11′E) as the research area. As a typical urbanized region in the Pearl River Delta, Shunde’s underground pipe network system has diverse characteristics: it includes combined drainage networks formed by historical evolution in urban villages (with widespread rainwater-sewage mixing), separate pipe networks supporting newly built residential communities and municipal roads (with separate rainwater and sewage pipes), and hidden drainage facilities such as dark ditches and culverts (influenced by flood control and space utilization needs in the Lingnan water town). The complex pipe network conditions provide a natural research carrier for analyzing sludge occurrence patterns. The on-site sampling work of urban pipe network sludge in this study strictly complied with the administrative management regulations for urban underground infrastructure in Guangdong Province and Foshan City. Prior to the on-site sampling, we had formally applied to the competent authority for permission to access the pipe network sites and collect samples, and obtained official approval documents from the Water-based City Development Headquarters Office of Ronggui Sub-district, Shunde District, Foshan City, Guangdong Province. This approval document explicitly authorized our research team to conduct non-destructive sampling operations at the 20 selected pipe network sites (covering urban villages, residential areas, municipal roads, and hidden ditches/culverts), and confirmed that the sampling process would not disrupt the normal operation of the urban drainage system nor cause environmental risks. All sampling activities were carried out under the supervision of the on-site management personnel of this competent authority to ensure compliance with safety and environmental protection requirements.

As shown in Table 1, to systematically reveal sludge characteristics under different pipe network scenarios, functional levels, and drainage systems, sampling was conducted in Shunde’s underground pipe networks following the principle of “full-scene coverage and functional differentiation,” with a total of 20 pipe network sludge samples collected.

**Table 1:** Distribution table of sampling types for pipe network sludge samples.

Serial Number	Sample ID	Sampling Type	Serial Number	Sample ID	Sampling Type
1	B-1	UV-Outlet-CF	11	R-1	Underground Ditch
2	B-2	UV-Outlet-CF	12	R-2	Underground Ditch
3	B-3	UV-Alley-CF	13	R-3	UV-Interception Pipe
4	B-4	RA-Outlet-Rainwater	14	R-4	UV-Sewage
5	B-5	UV-Outlet-CF	15	R-5	Underground Ditch
6	B-6	RA-Outlet-CF	16	R-6	Underground Ditch
7	B-7	UV-Outlet-CF	17	R-7	MD-Sewage Branch Pipe
8	B-8	UV-Outlet-CF	18	R-8	MD-Sewage Branch Pipe
9	B-9	MD-Secondary Trunk- Sewage	19	R-9	MD-Secondary Sewage Trunk
10	B-10	MR-Secondary Trunk-Sewage	20	R-10	Underground Ditch

**UV: Urban Village, RA: Residential Area, MD: Municipal Road, MR: Municipal Road,**

**CF: Combined Flow**

**Fig 1:**
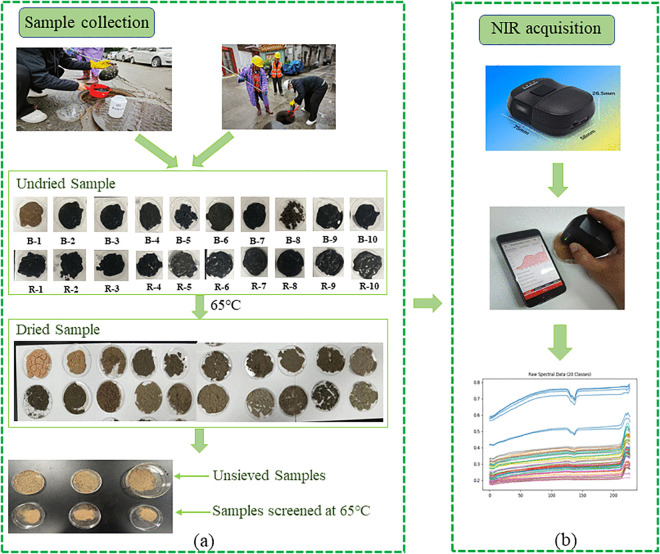
Collection of pipe network sludge samples; (a) Sample collection; (b) NIR acquisition.

The 20 collected pipe network sludge samples were dried at 65°C. This temperature setting can effectively remove moisture from the samples while minimizing the loss of volatile substances in the sludge, providing a stable basis for subsequent analytical tests. The drying process was continued until the sample mass remained constant to ensure complete evaporation of moisture. The dried sludge samples were sieved through a 100-mesh sieve to remove large particle impurities and foreign matter, ensuring the uniformity and representativeness of samples for subsequent analysis. The sieved samples were placed in clean polyethylene sealed bags, labeled with sample numbers, and stored in a desiccator to prevent moisture absorption from affecting the analysis results. The sample collection process is shown in Fig 1.

### 3.2. Basic physicochemical analysis

The basic physicochemical properties of pipe network sludge are core parameters reflecting its formation environment and occurrence state. Among them, pH is directly related to the sludge’s acid-base buffering capacity and pollutant speciation transformation potential, while electrical conductivity(EC) can characterize the total concentration of soluble ions in sludge [35].Together, they provide key basis for analyzing sludge differences among different pipe network types. To systematically reveal the basic physicochemical characteristics of sludge in the complex pipe network system of Shunde District, this study determined the pH and EC of all samples, with specific operating methods as follows:

#### 3.2.1. Determination of pH value.

Determination was performed using a PHS-3C pH meter (Shanghai Yidian Scientific Instruments Co., Ltd.). 10.00 ± 0.05 g of dried sludge sample was weighed into a 100 mL beaker, and 50 mL of deionized water was added (solid-liquid ratio 1:5). The mixture was stirred at 200 r/minfor 10 min using a magnetic stirrer and then allowed to stand for 30 min to achieve full mud-water stratification. The calibrated composite electrode was immersed in the supernatant, and the pH value was recorded after the reading stabilized. Two-point calibration with standard buffer solutions of pH = 4.00 and 6.86 was conducted before each measurement, and the electrode response slope was required to reach over 95%.

#### 3.2.2. Determination of EC.

Determination was conducted using a DDS-307A conductivity meter (Shanghai Leici Instrument Factory). The same supernatant used for pH measurement was adopted. A platinum black electrode was inserted into the solution, and the instrument performed automatic temperature compensation (reference temperature 25°C). The EC value was recorded after the reading stabilized. Calibration with 1413us/cm KCl standard solution was performed before each measurement, with the electrode constant required to be between 0.98 and 1.02.

#### 3.2.3. Near-infrared spectral analysis.

**Fig 2:**
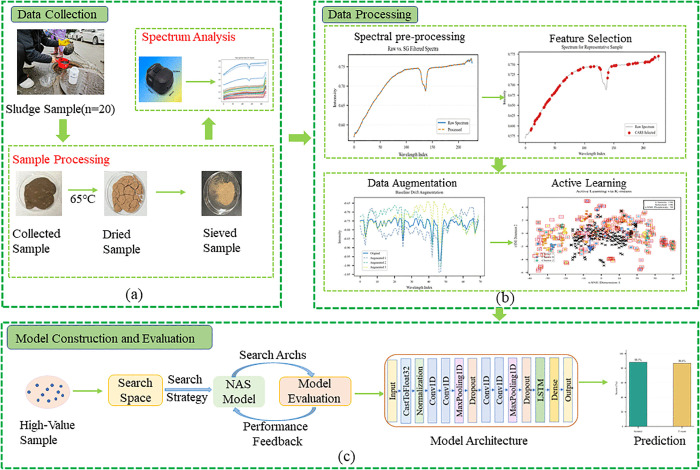
Collection of pipe network sludge samples and model construction.


To comprehensively explore effective detection methods for pipe network sludge, after collecting 20 sludge samples of different layout types, we chose to adopt NIR for the study. In this research, a handheld micro near-infrared spectrometer (model: NIR-S-G1, produced by Shenzhen Puyan Internet Technology Co., Ltd.) was used to collect spectral data of pipe network sludge. The near-infrared spectrometer used covers 228 wavelengths, with a spectral range of 900–1700 nm, a spectral interval of 3.89 nm, and a signal-to-noise ratio of 5000:1. The probe of the near-infrared spectrometer is aimed at the sludge. The light source emitted by the instrument is reflected back to the spectrometer through the sludge surface, and the reflected light is converted into a brightness value (BV) inside the spectrometer. A polytetrafluoroethylene compound was used to calibrate the portable micro near-infrared spectrometer. This compound has the highest reflectivity and the lowest absorptivity, ensuring that the device provides optimal results with minimal calibration errors. Before measuring each sludge sample, the spectrometer was calibrated with a standard white reference and a dark reference, and the BV of the white reference and dark reference were saved. The reflectance data of the pipe network sludge spectrum can be obtained through calculation Eq. (1), where I represents the brightness value of the sample, B represents the brightness value of the dark reference, and W represents the brightness value of the white reference. The entire experimental process is shown in Fig 2.

$$Refletance=\frac{I-B}{W-B}\times \text{1}\text{0}\text{0}\%$$ (1)

First, 20 original pipe network sludge samples were collected in this study, and 7 pieces of NIR spectral data were acquired for each sample, resulting in a total of 140 pieces of spectral data. After conducting systematic collation and in-depth analysis of this batch of spectral data, they were divided into a training set and a test set at a 7:3 ratio, with the corresponding sample sizes being 98 and 42 pieces respectively. To further optimize model performance, the training set was subjected to data augmentation using the baseline drift method, with an augmentation multiple set to 4. This ultimately yielded 392 valid spectral samples. This augmentation strategy not only fully covers the micro-heterogeneity of the same sludge sample but also effectively avoids model overfitting caused by excessive augmentation, achieving a balance between data utilization and model stability.

#### 3.2.4. Quality control and data correlation.

This study established a complete workflow of “Quality Control (QC) - Stepwise Preprocessing” and conducted the determination of key physicochemical indicators (pH and EC) using standard laboratory techniques, with specific details as follows: during the QC phase, interference was avoided at the source—on one hand, to address moisture content differences in sludge from different sources, all samples were first dried to absolute dryness at 65°C and then sieved through a 100-mesh nylon sieve to standardize particle size, which eliminated the interference of “moisture scattering and particle blocking” on spectral absorption signals and ensured a consistent matrix environment for all samples; on the other hand, a constant temperature and humidity device was used to maintain a stable detection environment throughout the experiment (temperature at 25 ± 1°C, humidity at 50 ± 5%), while the spectrometer’s built-in environmental sensors recorded real-time temperature and humidity data, avoiding “spectral baseline shifts caused by environmental water vapor absorption and temperature fluctuations” and ensuring the stability of raw spectral data. During the stepwise preprocessing phase, residual interference was specifically eliminated: first, a 7-point window Savitzky-Golay (SG) smoothing method (with 2nd-order polynomial fitting) was adopted to filter out high-frequency fluctuations caused by the instrument’s electronic noise and sample particle scattering, preserving the characteristic absorption signals in the spectrum; subsequently, the adaptive iteratively reweighted penalized least squares method was used to eliminate two issues—”slow baseline drift caused by long-term use of the light source and scattering-induced baseline shifts due to uneven sludge surfaces”—further improving the spectral data’s signal-to-noise ratio. Meanwhile, pH was measured via potentiometry in accordance with GB/T 6920−1986, and EC was determined using a conductivity meter following GB/T 11277−2008. Multiple quality control measures were implemented to ensure measurement accuracy and precision: the pH meter was calibrated daily with standard buffer solutions (pH 4.00, 6.86, 9.18), the conductivity meter was calibrated with a 0.01 mol/L KCl standard solution (EC = 1413 us/cm at 25°C), and all chemical analyses were performed in triplicate parallel experiments (results expressed as the mean, with relative standard deviation controlled within 5%), fully guaranteeing the reliability of determined data. For subsequent analysis, the measured basic physicochemical indicators (e.g., pH, EC) will be statistically analyzed in combination with different pipe network types to reveal differences in sludge physicochemical characteristics under various scenarios, providing basic data support for analyzing pipe network sludge occurrence patterns; meanwhile, NIR data will be independently used to analyze the diversity of sludge spectral characteristics and their correlation with pipe network functional levels and drainage systems.

### 3.3. Spectral preprocessing and modeling methods

After sample collection, preliminary data collation and analysis, and determination of corresponding basic physicochemical indicators, we obtained abundant raw spectral data of pipe network sludge. However, raw data often have various issues. These near-infrared spectral data not only contain sample information but also interfere with information such as background and noise, which can affect the final modeling accuracy. To correct baseline drift, reduce spectral scattering, eliminate random noise, and improve the signal-to-noise ratio [36], this study adopted the following preprocessing methods for the pipe network sludge spectral data: Savitzky-Golay (SG) smoothing, multiplicative scatter correction (MSC), standard normal variate (SNV), first derivative (FD), second derivative (SD), orthogonal signal correction (OSC), logarithmic transformation (LG), and two hybrid preprocessing methods (SG smoothing combined with MSC (SG + MSC), and SG smoothing combined with SNV (SG + SNV)) [37].

Among them, SG uses a polynomial to fit data within a moving window, improving the signal-to-noise ratio by reducing noise. The SG spectrum is calculated via Eq. (2), where $x_{k+\text{1}}$ represents the discrete spectrum, H is the normalization factor, w denotes the smoothing window radius, and $h_{i}$ is the smoothing coefficient.


$$x_{k}=\frac{\text{1}}{H}\sum_{-w}^{+w}x_{k+\text{1}}h_{i}\;\;$$ (2)

FD and SD are often used to eliminate interference from baselines and other backgrounds to improve sensitivity. For discrete spectrum $x_{k}$, the first derivative spectrum and second derivative spectrum at wavelength k with a difference width of g are calculated according to Eq. (3) and Eq. (4), respectively.


$$x_{k}=\frac{x_{k+g}-x_{k-g}}{g}\;\;$$ (3)

$$x_{k}=\frac{x_{k+g}-\text{2}x_{k}+x_{k-g}}{g^{\text{2}}}\;$$ (4)

SNV can be used to eliminate the influence of solid particle size and optical path changes on near-infrared diffuse reflectance spectra. The procedure of this method involves calculating the mean of the discrete spectrum, subtracting the mean spectrum from the original spectrum, and finally dividing by the standard deviation of the bands. Its resulting spectrum is calculated via Eq. (5), where $x_{k}$ is the discrete spectrum, $\overset{―}{x}$ is the mean spectrum, and m is the total number of spectral data points.


$$x_{k}=\frac{x_{k}-\overset{―}{x}}{\sqrt{\frac{\sum_{k-\text{1}}^{m}{\left(x_{k}-\overset{―}{x}\right)}^{\text{2}}}{m-\text{1}}}}\;\;$$ (5)

MSC, similar to SNV, is used to eliminate scattering effects caused by uneven particle distribution. For the spectrum $x_{k}$, the mean is taken as its ideal spectrum. Linear regression is performed between the original spectrum and the ideal spectrum to obtain b and $b_{\text{0}}$, followed by linear transformation. The specific formula is shown in Eq. (6).


$$x_{k}=\frac{x-b_{\text{0}}}{b}\;\;$$ (6)

LG involves taking the logarithm of raw spectral data to calculate the spectral absorbance, as shown in Eq. (7).


$$x_{k}=\frac{\text{1}}{Refletance}\;$$ (7)

OSC was first proposed by Svante Wold in 1998. Its core idea is to remove information in the data matrix X that is irrelevant to the target variable Y through mathematical orthogonalization, thereby improving the predictive performance of the model.

After preprocessing, the quality of spectral data is improved. However, to accurately extract relevant information about pipe network sludge from these optimized data, selecting appropriate analytical models is crucial. Therefore, in this paper, SVM, Random Forest (RF), and XGBoost models are used as representatives of ML, while neural network models based on the NAS framework and multi-layer perceptrons(MLP) are used as representatives of DL [38,39]. SVM is a widely used classification model, first proposed by Dr. Cortes and Dr. Vapnik, which performs classification or regression analysis by finding the optimal separating hyperplane in a high-dimensional space [40]. RF is an ensemble learning method, first proposed by Dr. Breiman, which improves the accuracy and stability of classification or regression by constructing multiple decision trees and combining their prediction results [41]. XGBoost is an efficient gradient boosting decision tree (GBDT) algorithm proposed by Chen and Guestrin, which enhances the model’s accuracy and training speed through optimized computation and regularization techniques [42].

Among them, representative DL models include the combination of CNN and LSTM neural networks based on the NAS architecture, as well as MLP. MLP is a feedforward artificial neural network composed of an input layer, hidden layers, and an output layer [43,44]. It adjusts network weights through backpropagation algorithms to learn data features, possessing strong nonlinear fitting capabilities [45]. NAS is an automated neural network architecture search method that automatically explores and optimizes neural network structures through algorithms to find the optimal model architecture. It mainly includes search space, search strategies, and performance evaluation strategies. The search space is a collection of all candidate neural network architectures; a certain search strategy is used to select the optimal network structure from it, and then a certain metric is used to evaluate its performance to determine whether the structure is superior or inferior, thereby guiding the search strategy for re-search [46]. NAS significantly reduces the time and resources required for manual design and adjustment of model structures. In the research on classification and identification of pipe network sludge, the advantages of NAS architecture neural networks are fully demonstrated. By setting a reasonable search space covering various network structures that may be suitable for spectral feature extraction and classification tasks of pipe network sludge, and then using efficient search strategies such as reinforcement learning and evolutionary algorithms, potential high-quality architectures suitable for current data can be quickly screened out.

### 3.4. CABNas-nir method

**Fig 3:**
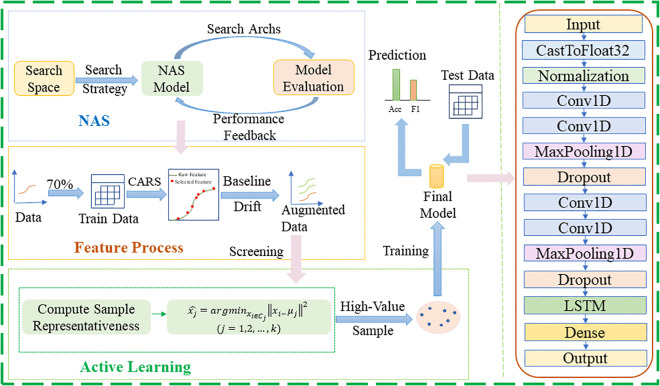
Flowchart of the CABNas-nir algorithm.

After the aforementioned exploration of spectral preprocessing and modeling methods, to further improve model performance and achieve more accurate analysis, we innovatively propose the CABNas-nir method. Essentially, CABNas-nir is a CNN+LSTM neural network model built under the NAS framework, integrating the CARS algorithm, baseline drift augmentation, and K-means clustering-based AL algorithm. The specific schematic diagram of the algorithm is clearly shown in Fig 3.

To better describe the specific process of integrating the CARS algorithm, baseline drift augmentation, and AL algorithm, we set the following relevant parameters. Let the original spectral dataset be $D=D_{train}\cup D_{test}$,where the training set $D_{train}=\{(X_{i},y_{i}{\}}_{i=\text{1}}^{N}$($X_{i}\in R^{d}$ denotes d-dimensional spectral samples, $y_{i}\in \{\text{1},\text{2},\ldots ,C\}$ represents the corresponding class labels, and N is the total number of training samples), and the test set $D_{test}=\{(X_{j},y_{j}{\}}_{j=\text{1}}^{M}$ (M is the total number of test samples). For the CARS algorithm, the number of iterations is set as $T_{CARS}$, and the number of cross-validation folds as $K_{CV}$. For baseline drift enhancement, the spline interpolation control point sets are set as $X_{ctrl}=\{x_{\text{1}}^{c},x_{\text{2}}^{c},\ldots ,x_{L}^{c}\}$and $y_{ctrl}=\{y_{\text{1}}^{c},y_{\text{2}}^{c},\ldots ,y_{L}^{c}\}$ (L is the number of control points), with the interpolation type as kind∈{linear,cubic}; For the K-means AL sampling strategy, the number of clusters is set as $K_{clus}$.

First, CARS feature selection is performed on the preprocessed training data $D_{train,scaled}=\{X_{i}^{scaled},y_{i}{\}}_{i=\text{1}}^{N}$ (where $X_{i}^{scaled}$ denotes standardized samples). Its core logic is to iteratively optimize the feature subset: in the t-th iteration($t=\text{1},\text{2},\ldots ,T_{CARS}$), the number of retained features is $K_{t}=\text{max}(\text{2},\left\lfloor {\left(\frac{\text{1}}{t+\text{1}}\right)}^{\text{0}.\text{3}}\right\rfloor )$(where ⌊·⌋ is the floor function); based on the feature subset $R_{t-\text{1}}$ retained in the previous iteration, a partial least squares model $PLS_{t}=F_{PLS}(X_{train,scaled}\left[:,R_{t-\text{1}}\right],y_{train})$ is trained (where $F_{PLS}$ represents the PLS fitting function, $X_{train,scaled}$ is the standardized training sample matrix, and $y_{train}$ is the training label vector); a new feature subset $R_{t}$ is obtained through weight coefficient screening. Finally, the optimal feature subset $R^{*}=\text{arg}{min}_{t}Loss\left({PLS}_{t}\right)$, where Loss(·) is the model loss function, and the corresponding training set after feature selection is $D_{train,CARS}={\left\{X_{i}^{scaled}[:,R^{*}],y_{i}\right\}}_{i=\text{1}}^{N}$.

Subsequently, baseline drift enhancement is performed on $D_{train,CARS}$. Define the baseline drift signal generation function as $b\left(\cdot ;X_{ctrl},y_{ctrl},kind\right)=L_{line}\left(\cdot ;X_{ctrl},y_{ctrl},kind\right),$ where $L_{line}$ is a spline interpolation function. The augmented sample for a single sample can be expressed by Eq. (8). Let the augmentation multiple be $K_{aug}$. Then, the augmented training set is $D_{train,aug}={U\;}_{k=\text{1}}^{K_{aug}}{\left\{X_{i}^{aug,k},y_{i}\right\}}_{i=\text{1}}^{N}$, where $X_{i}^{aug,k}$ denotes the sample generated in the k-th augmentation.


$${X\;}_{i}^{aug}={X\;}_{i}^{CARS}+b\left(\cdot ;X_{ctrl},y_{ctrl},kind\right)$$ (8)

Next, a diversity-oriented AL strategy integrated with K-means clustering was implemented on the augmented training set$\;D_{train,aug}$. This strategy belongs to the “diversity priority” paradigm in AL, which aims to screen representative samples that cover key feature regions of the data distribution while avoiding information redundancy. The specific integration process and parameter optimization are detailed as follows:First, to eliminate the interference of feature dimension differences (e.g., varying spectral intensity ranges) on K-means clustering results, the spectral feature samples in $D_{train,aug}$ were preprocessed with Z-score standardization (i.e., each feature was transformed to have a mean of 0 and a standard deviation of 1). Subsequently, cluster partitioning $\left\{C_{\text{1}},C_{\text{2}},\ldots ,C_{K_{clus}}\right\}$ was obtained by applying the K-means algorithm $\kappa (\cdot ;K_{clus})$, with hyperparameters set as max_iters=300 (to ensure convergence) and random=42 (to guarantee result reproducibility).

To avoid subjective selection of the number of clusters, this study adopted a two-step quantitative evaluation framework to determine the optimal number of clusters, $K_{clus}$: first, by comprehensively considering the number of original pipe network sludge sample types (20), the size of the augmented sample set (392), and the micro-variability of spectral data (e.g., subtle differences in characteristic absorption peaks of C-H/N-H bonds among different sampling points of the same functional scenario), the range of the number of clusters K was adjusted to 20–100. This range setting balances two core demands: on one hand, the lower limit of 20 corresponds to the number of original sample types, ensuring that each original sample type can be covered by at least one cluster to avoid missing key source characteristics; on the other hand, the upper limit of 100 prevents excessive clustering fragmentation (which would lead to redundant marked samples and increased labeling costs) while adapting to the fine-grained distribution of 392 augmented spectra (generated by 4-fold baseline drift augmentation, containing rich micro-variation information such as instrument precision fluctuations and environmental humidity interference).

Specifically, in the first step, a preliminary screening was conducted using the Elbow Method, where the Within-Cluster Sum of Squares (WCSS) corresponding to each K value in the range of 20–100 was calculated. The WCSS values showed a clear trend: when K<30, WCSS decreased sharply (e.g., WCSS = 15.2 at K=20, WCSS = 11.8 at K=25, WCSS = 8.3 at K=30, indicating that increasing the number of clusters can effectively reduce the intra-cluster data dispersion; when K>30, the decline rate of WCSS slowed significantly (e.g., WCSS = 8.1 at K=35, WCSS = 7.9 at K=40, WCSS = 7.7 at K=100, meaning that continuing to increase clusters no longer significantly improves the concentration of intra-cluster data. Thus, the “elbow point”—where the WCSS shifts from a sharp decline to a flat trend—was identified as the preliminary candidate number of clusters, which corresponded to K=30 in this study.

On this basis, the second step involved verifying the clustering quality using the Silhouette Coefficient (ranging from −1–1, with values closer to 1 indicating better clustering quality), a metric that quantifies a sample’s similarity to its own cluster (cohesion) and its dissimilarity to other clusters (separation). The results showed that the candidate number of clusters K=30 achieved the highest Silhouette Coefficient (0.75), significantly outperforming adjacent K values (e.g., 0.68 when K=25, 0.63 when K=35, 0.58 when K=20, and 0.45 when K=100. This indicates that at K=30, the intra-cluster spectral samples have high similarity (e.g., spectra of the same original sample type after augmentation are clustered together, with consistent absorption peak positions at 1600–1700 nm), while inter-cluster samples have significant differences (e.g., spectra of urban village combined-flow sludge and municipal road sewage branch pipe sludge are clearly separated, matching the spectral intensity differences shown in Fig 6(a)). As a result, K=30 was finally confirmed as the optimal $K_{clus}$.

**Fig 4:**
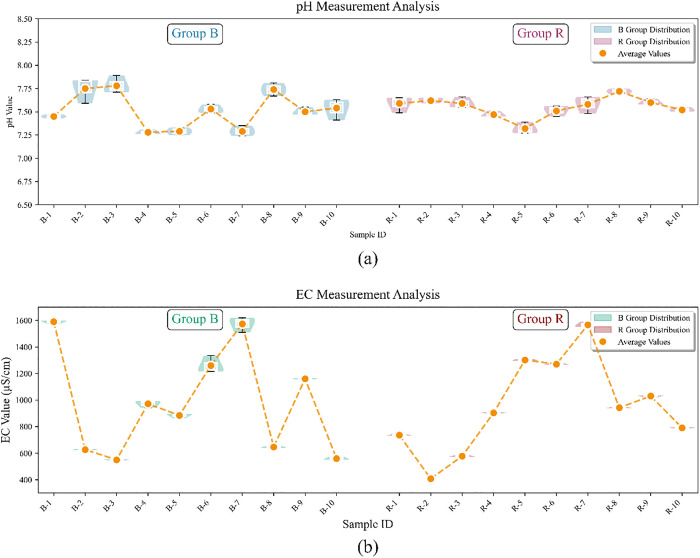
Results of physicochemical values of pipe network sludge: (a) Results of pH values; (b) Results of EC values.

**Fig 5:**
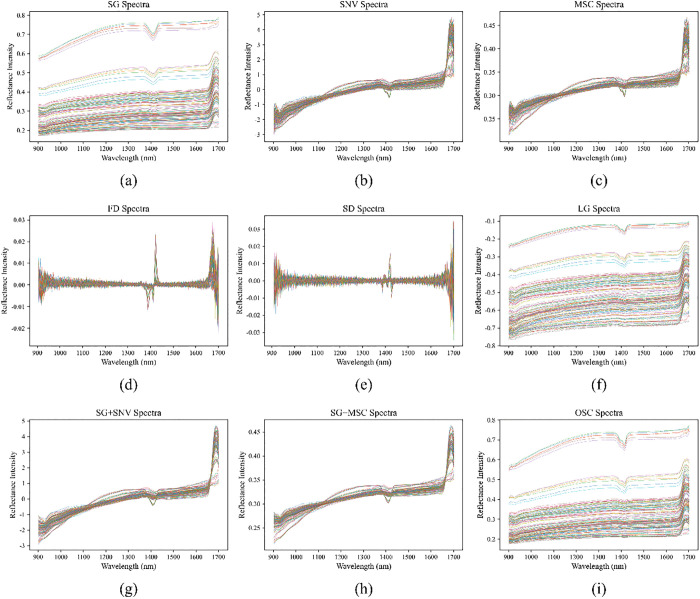
Preprocessing spectrograms: (a) SG spectrogram; (b) SNV spectrogram; (c) MSC spectrogram; (d) FD spectrogram; (e) SD spectrogram; (f) LG spectrogram; (g) SG + SNV spectrogram; (h) SG + MSC spectrogram; (i) OSC spectrogram.

After clustering, the center of the c-th cluster $C_{c}$ was computed as $\mu_{c}=\frac{\text{1}}{\left|C_{c}\right|}\sum_{X\in C_{c}}X\;$,where $\left|C_{c}\right|$ denotes the number of samples in cluster $C_{c}$ — and from each cluster, the sample closest to the cluster center was selected as the representative sample, with its index satisfying $i_{c}=\text{arg}{min}_{X\in C_{c}}{∥X-\mu_{c}∥}_{\text{2}}$ (where ${∥\cdot ∥}_{\text{2}}$ is the L2 norm, i.e., Euclidean distance); to ensure the selected samples truly represent the underlying data distribution, two validation steps were added: first, an intra-cluster consistency check, where the within-cluster variance of the selected samples was calculated, and all values were less than 0.15 (consistent with the variance of the original training set) — this confirmed that the selected samples retained the core feature characteristics of each cluster; second, an inter-cluster diversity check, where the pairwise Euclidean distance between cluster centers of the selected samples was measured, and all inter-cluster distances exceeded 2.0 — this ensured sufficient feature differences between representative samples to avoid redundancy, and the training set after AL screening was thus defined as $D_{train,selected}={\left\{X_{i_{c}}^{aug},y_{i}\right\}}_{c=\text{1}}^{K_{clus}}$.

Finally, the optimal neural network model was constructed via the NAS framework. First, samples in $D_{train,selected}$ were reshaped into $X_{reshape}\in R^{\text{1}*f}$ (where $f=|R^{*}|$ is the post-feature-selection dimension) is the post-feature-selection dimension). The neural network architecture employed herein is a hybrid structure integrating CNN and LSTM, which is specifically designed to match the sequential characteristics and local feature attributes of near-infrared spectral data—CNN is responsible for extracting fine-grained local spectral features (such as characteristic absorption peaks of functional groups like C-H and N-H bonds), while LSTM captures long-range dependencies between adjacent wavelength bands, and the two components complement each other to enhance the model’s ability to represent spectral features comprehensively. Specific details of the NAS implementation are as follows: Search Space: Centered on the above-mentioned CNN-LSTM hybrid architecture, covering key network components and their parameter ranges, including Conv1D layers (filters: 8–64, kernel sizes: 3/5/7, activation function: ReLU), LSTM layers (units: 16–128, activation function: tanh), and Dropout layers (fixed rate: 0.3, used to suppress overfitting without excessively losing valid feature information); Hyperparameter Optimization: Adopted a differentiable search strategy, with the optimization objective set to minimize the cross-entropy loss of the pipe network sludge multi-classification task, and hyperparameters (including the number of filters and kernel sizes in Conv1D layers, as well as the number of units in LSTM layers) were iteratively adjusted through gradient descent algorithm to ensure the model converged to the optimal parameter combination; Computational Resources: The experiments were conducted on an NVIDIA GeForce RTX 3050 GPU, with training hyperparameters configured as epochs = 800 (to ensure the model reached sufficient convergence, as verified by the stable trend of training and validation loss curves) and max search trials = 20 (to balance the efficiency of architecture search and the quality of the final optimal model). The optimal model $C^{*}=(A,S,D_{train,selected})$ was obtained by searching the predefined space S using the optimization algorithm A. This model fully integrates the advantages of feature selection (screening key spectral bands via CARS), data augmentation (expanding samples via baseline drift), and sample screening (selecting high-value samples via AL), thereby realizing accurate classification of pipe network sludge sources.

In summary, the pseudocode of the CABNas-nir algorithm is summarized as follows:

**Algorithm 1:** CABNas-nir: A Near-Infrared Classification for Urban Pipe Network Sludge on the Fusion Algorithm of NAS Framework and Active Learning

**Input:** Spectral dataset file pat $\left(loads\;D=D_{train}\cup D_{test}\right);$ CARS parameters: $T_{CARS},K_{CV},$ Baseline drift augmentation parameters:$X_{ctrl}$,$Y_{ctrl},kind$; K-means AL parameters: $K_{clus}$

**Output**: Optimized classification model C,evaluation metrics:{Accuracy,Precision,Recall,F1}

1:Initialize CARS model:$R^{*}=\emptyset (optimal\;feature\;set),Loss_{min}=+\infty (\text{min}PLS\;loss)$

2:**for** $t=\text{1}\;to\;T_{CARS}\;do$

   $k_{t}=max(\text{2},\left\lfloor {\left(\frac{\text{1}}{t+\text{1}}\right)}^{\text{0}.\text{2}}\right\rfloor )$

   $PLS_{i}=F\_PLS(X_{train_{scaled}}[:,R_{prev}],y_{train},K_{CV})$

   **End for**

$D_{train\_CARS}=\{\left({X_{i}}^{scaled\left[:,R^{*}\right]},y_{i}\right\}\_{\left\{i=\text{1}\right\}}^{N}$

3:**While** $D_{train\_aug}=D_{train\_CARS}$ **do**

   **For** $k\in \{\text{1},\ldots ,k_{aug}\}$ **do**

    **For** each(Xi^CARS,yiin Dtrain_CARS: **do**

     Xi^aug=Xi^CARS+b(Xi^CARS;Xctrl,Yctrl,kind)

     $add(X_{i}^{aug},y_{i}\{t\}o\;D_{train\_aug}$

**End for**

    $D_{train\_aug}=D_{train\_aug}\cup D_{aug}$

4: **While** $X_{train\_aug}\in R^{N\times f}$ **do**

    $Cluster=KMeans\left(n_{clusters}=k_{clust}\right).fit(X_{aug})$

 **For** c∈{$\text{1},\ldots ,k_{clust}$}

    $X_{c}=\{X_{i}|X_{i}\in X_{aug},Cluster.labels_{i}=c\}$

    selected_idx=selected_idx∪{closest_idx}

**End for**

    $D_{train\_selected}=\{(X_{aug[i]},y[i])|i\in selected\_idx\}$

5: **While** $X_{train\_reshaped}\in R^{N\times 1\times f}$ **do**

    $Conv=Conv\text{2}D/Conv\text{1}D(filters\in \left\{\text{8},\ldots ,\text{64}\right\},kernel_{size}\in \left\{\text{3},\text{5},\text{7}\right\})$

    Rnn={LSTM(units∈{16,…,128},return_sequences∈{True,False}}

    ClassHead={Dense(C,activation=softmax)

 **End for**

    $C^{*}=NAS\_Search(X_{reshaped},y_{selected},SearchSpace)$

 **End while**

**Return Metrics:**(Accuracy,Precision,Recall,F1)

    **Model** $C=C^{*}$

For the feature selection module, this study selects four representative algorithms: CARS, particle swarm optimization (PSO), genetic algorithm (GA), and simulated annealing (SA). These algorithms differ significantly in their ways of selecting key features: CARS is based on a competitive adaptive reweighting mechanism. It iteratively calculates the weight coefficients of feature variables, retains features with higher weights (simulating the evolutionary logic of “survival of the fittest”), gradually eliminates redundant information, and finally screens out sensitive bands strongly related to the classification task [47]. PSO simulates the group cooperation behavior of bird flocks foraging. It treats each feature subset as a “particle” and searches for the globally optimal feature combination through information sharing among particles and position updates (iteratively optimizing speed and position parameters) [48]. GA draws on the principle of natural selection in biological evolution. It encodes feature subsets as “chromosomes” and realizes iterative optimization of feature subsets through selection, crossover, and mutation operations, retaining feature combinations with higher fitness [49]. SA originates from the physical annealing process. It gradually reduces the probability of accepting worse solutions by controlling temperature parameters, slowly searches for the optimal feature subset in the global range, and effectively avoids falling into local optima [50].

After determining the feature selection module, the screened features need to be further expanded in sample size through data augmentation. Data augmentation is a technique that generates “virtual samples” by applying reasonable physical or algorithmic transformations to original samples, aiming to alleviate the problem of biased feature distribution in small-sample scenarios and improve the generalization ability of the model [51]. In this study, five methods are adopted: Gaussian noise, spectral smoothing, wavelength shifting, baseline drift, and intensity scaling. Gaussian noise simulates random noise interference in the instrument detection process by adding random perturbations conforming to a Gaussian distribution to spectral data [52,53]. Spectral smoothing is based on polynomial fitting within a sliding window (e.g., SG smoothing), reducing the interference of high-frequency noise on features while retaining the overall spectral trend. Wavelength shifting simulates peak position shifts caused by instrument wavelength accuracy fluctuations or changes in detection environment by slightly shifting feature wavelengths (±1–3 nm). Baseline drift reproduces the slow drift of spectral baselines caused by factors such as ambient temperature and humidity, and pipe material adsorption by generating slowly changing baseline signals through spline interpolation and superimposing them on original spectra, which is a highly suitable augmentation method for the spectral characteristics of pipe network sludge. Intensity scaling simulates signal strength changes caused by light intensity fluctuations or sample concentration differences by adjusting spectral intensity values proportionally (±5%−10%).

After determining the feature selection and data augmentation modules, AL is employed to further screen high-value samples. AL is a learning paradigm that allows models to proactively select the most informative samples for labeling during training, maximizing model performance improvement under limited labeling costs. Mainstream AL sampling strategies include uncertainty sampling, diversity sampling, query by committee (QBC), consistency-based sampling, and temporal output difference (TOD): Uncertainty sampling focuses on samples with the lowest model prediction confidence. Among them, minimum confidence sampling selects samples with the lowest confidence in the highest predicted probability class, while maximum entropy sampling chooses samples with the highest entropy in the predicted probability distribution (higher entropy indicates richer information diversity) [54]. Diversity sampling maximizes differences between samples (e.g., using maximum distance selection based on cosine similarity or Euclidean distance) to ensure selected samples cover different regions of the feature space and avoid information redundancy [55]. QBC trains multiple heterogeneous models (such as SVM, RF, and MLP) to form a “decision committee” and selects samples with the greatest prediction divergence among models. Higher divergence indicates greater value of samples for model optimization [56]. Consistency-based sampling compares model predictions between samples and their augmented versions (e.g., with added noise or slight transformations) to select samples with higher prediction inconsistency [57]. TOD evaluates sample uncertainty by calculating output differences of the same sample between two consecutive learning cycles, with samples showing larger differences prioritized for labeling [58].

The selection of CARS, Baseline Drift Correction, and K-means Clustering algorithms is a tailored solution addressing the unique challenges in NIR spectral classification of urban pipeline sludge, with each algorithm specifically breaking through core bottlenecks in task processing. In the feature selection stage, CARS iteratively calculates the weight coefficients of spectral bands via PLS regression, simulating a “survival-of-the-fittest” mechanism to retain spectral bands highly correlated with sludge source differences (e.g., the 1400–1700 nm band corresponding to C-H/N-H/O-H bond vibrations). Simultaneously, it eliminates redundant noise such as instrument background interference, perfectly adapting to the characteristics of high dimensionality and strong redundancy in NIR data, and ensuring only “source-discriminating spectral fingerprints” are retained. In the data augmentation stage, Baseline Drift Correction can simulate real physical variations in NIR detection (e.g., temperature and humidity fluctuations, adsorption effects of pipeline materials, and baseline shifts caused by instrument precision drift). Unlike random augmentation methods such as Gaussian noise, this technique generates physically meaningful baseline signals through spline interpolation and superimposes them onto the key feature bands filtered by CARS. While preserving the positions and intensity ratios of characteristic peaks, it expands the sample size, effectively addressing the challenges of small sample size and biased feature distribution in sludge datasets. In the AL phase, K-means Clustering exhibits a particularly prominent advantage in “diversity-prioritized sampling”: the algorithm clusters the expanded samples based on spectral feature similarity, selecting representative samples close to cluster centers to avoid information redundancy. Considering the quality variations among expanded samples, K-means ensures the model focuses on high-value samples that cover core feature regions, improving the model’s generalization ability without increasing labeling costs. These three methods form a tight collaborative chain: CARS provides accurate feature objects for Baseline Drift Correction, Baseline Drift Correction enriches the sample pool for the AL stage, and K-means further refines sample quality. This collaborative mechanism fully meets the core requirements of addressing spectral redundancy, small sample size, and complex variations in sludge sources in the task.

In subsequent studies, we systematically verified the performance differences among various feature selection algorithms, data augmentation methods, and AL strategies through comparative experiments (see Section 4.3 for details). These experimental results not only confirm the optimal adaptability of CARS, baseline drift, and K-means clustering-based AL but also provide data support and direction guidance for the further optimization of the CABNas-nir framework.

### 3.5. Evaluation metrics

After completing sample collection, data analysis, determination of basic physical and chemical indicators, and construction of the CABNas-nir model through spectral preprocessing and modeling methods, we need to evaluate the model performance. Four evaluation metrics are used: accuracy (Eq. 9), precision (Eq. 10), recall (Eq. 11), and F1-score (Eq. 12). Among them, TP represents true positives, i.e., the number of samples that are actually positive and predicted as positive by the model; TN is true negatives, i.e., the number of samples that are actually negative and predicted as negative by the model; FP is false positives, i.e., the number of samples that are actually negative but predicted as positive by the model; FN is false negatives, i.e., the number of samples that are actually positive but predicted as negative by the model. Generally, both accuracy and F1-score need to be considered when evaluating the model performance.


$$Accuracy=\frac{TP+TN}{TP+TN+FP+FN}$$ (9)

$$Precision=\frac{TP}{TP+FP}\;$$ (10)

$$Recall=\frac{TP}{TP+FN}$$ (11)

$$F\text{1}=\frac{\text{2}\times Precision\times Recall}{Precision+Recall}$$ (12)

In the specific context of pipe network sludge source tracing, the cost of False Positives (misidentifying a pollution source) and False Negatives (missing a pollution source) are both high. Therefore, while we present four metrics, Accuracy and F1-score are considered the primary evaluation metrics in this study. Accuracy provides an intuitive measure of overall correctness, while F1-score offers a robust assessment by balancing Precision and Recall, ensuring the model’s reliability even when class boundaries are complex.

## 4. Experiments and results

### 4.1. Analysis of basic physicochemical index results

As core physicochemical indicators reflecting acid-base properties and soluble ion concentration, the pH value and EC of pipe network sludge are directly related to the pollutant forms, biological activity, and environmental behavior of sludge. Combined with the distribution characteristics of pipe network sludge sampling types in Table 1 (Section 3.1), a cross-analysis of the determination results in Fig 4 shows the following: The average pH of 20 pipe network sludge samples ranges from 7.28 to 7.78, showing an overall weakly alkaline property (pH > 7), which is consistent with the alkaline background characteristics of soil and water in the Pearl River Delta region. The average EC ranges from 408.00 to 1590.00 us/cm with significant differences, indicating that the content of soluble substances in sludge from different pipe network types varies greatly. Specifically, among the urban village samples, the pH of “Urban Village-Outlet-Combined Flow” fluctuates greatly (7.29–7.75); sample B-3 of “ Urban Village -Alley- Combined Flow “ has the highest pH (7.78); the EC is generally high, with B-1 and B-7 reaching 1590.00 and 1573.33 us/cm, which is presumably related to the mixing of combined sewage and high salt content in domestic sewage. Among the residential area samples, sample B-4 of “ Residential Area-Outlet-Rainwater” has the lowest pH (7.28) and an EC of 972.00 us/cm, both lower than those of B-6 of “ Residential Area-Outlet-Combined Flow”, which may be related to acidic substances and surface minerals brought by rainwater. The pH (7.50–7.54) and EC (942.50–1030.00 us/cm) of municipal road samples are relatively stable, reflecting the homogenization effect of large-scale pipe networks. The average pH of hidden ditches and culverts is 7.50 with small fluctuations (7.32–7.62); in terms of EC, R-2 is the lowest (408.00 us/cm), while R-5 and R-6 are higher (1301.67 and 1270.00 us/cm), reflecting the stability of the closed environment and the impact of local pollution sources. In summary, the distribution differences of pH and EC reveal the strong correlation between the physicochemical properties of pipe network sludge and pipe network functions as well as drainage systems, providing an important physicochemical basis for subsequent near-infrared spectroscopy-based identification of origin [59].

### 4.2. Preprocessing methods and spectral analysis

To improve the quality of spectral data for pipe network sludge samples, based on prior knowledge of spectral analysis characteristics, this study initially selected nine candidate pretreatment methods, namely SG, SNV, MSC, FD, SD, LG, SG + SNV, SG + MSC, and OSC. Subsequently, a quantitative strategy was employed to explore the most suitable pretreatment method for the data in this paper.. The spectral diagrams of each pretreatment method are shown in Fig 5.

**Fig 6:**
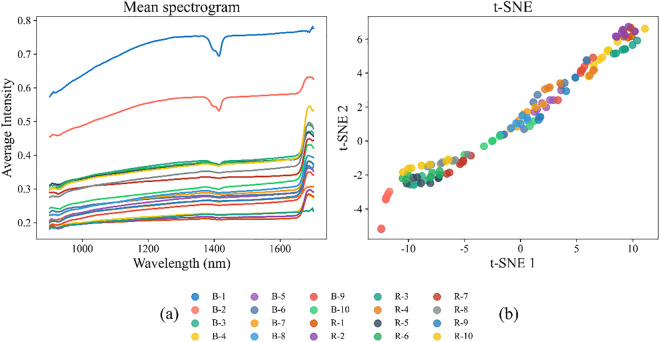
Spectral analysis diagram of pipe network sludge samples: (a) Average spectrogram; (b) t-SNE diagram.

Through a series of preprocessing of spectral data, we have established a clearer basis for analyzing the spectral characteristics of sludge samples. Based on the preprocessed data, we further explored the spectral properties of sludge samples from a visualization perspective: The average spectra of pipe network sludge samples of different sampling types are shown in Fig 6(a). It can be seen that although the sludge from different pipe network areas such as urban villages, municipal roads, and hidden ditches has highly similar overall variation trends in the near-infrared band of 900–1700 nm (e.g., the absorption peaks and trends are consistent with increasing wavelength), there are significant differences in spectral intensity. Specifically, the spectral intensity of urban village-outlet-combined flow samples (B-1, B-2, etc.) is significantly higher than that of hidden ditch samples (R-1, R-2, etc.), which intuitively reflects the differences in the contents of chemical components such as organic matter and inorganic salts in the two types of pipe network sludge (consistent with the EC value analysis in Section 4.1: samples with high EC correspond to higher spectral absorption, due to the enhanced light scattering/absorption by soluble ions). To identify complex non-linear patterns in the high-dimensional spectral data, t-distributed Stochastic Neighbor Embedding (t-SNE) was employed for visualization. The results of t-SNE are shown in Fig 6(b), which further verify the correlation between spectral characteristics and pipe network functions: Samples show an obvious clustering trend in the 2D t-SNE space.—urban village combined flow samples and hidden ditch samples form relatively independent clusters respectively, while municipal road samples gather in the middle area. [60] This distribution indicates that sludge from the same type of pipe network has highly similar spectral “fingerprints” due to long-term influence of similar sewage components and hydraulic conditions; in contrast, sludge from pipe networks with different functions has significantly distinct spectral characteristics due to differences in pollutant sources (domestic sewage/rainwater/mixed sewage) and migration paths. In summary, NIR can effectively capture the differences in chemical compositions of pipe network sludge, and it is feasible to realize effective classification and identification of pipe network sludge using NIR.

### 4.3. CABNas-nir modeling method for pipe network sludge

After a series of pretreatment operations on pipe network sludge samples, to improve the accuracy of classification and identification, we designed the following experiments:

(1) Comparative experiments on cross combinations of feature selection methods and model architectures
(2) Comparative experiments on different data augmentation methods
(3) Comparative experiments on different AL sampling strategies

**Fig 7:**
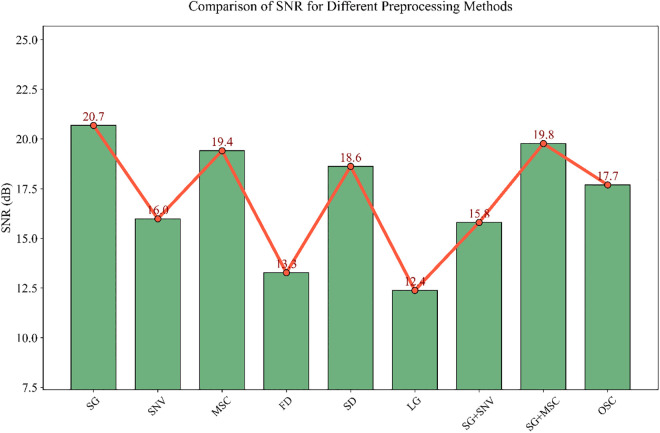
Comparative analysis of SNR after preprocessing methods.

After a series of pretreatment operations on pipe network sludge samples, to accurately screen out the pretreatment method suitable for the characteristics of spectral data, this study quantitatively evaluated the pretreatment effect through signal-to-noise ratio (SNR). A higher SNR indicates better separation between effective spectral signals and noise after pretreatment, and the data quality is more conducive to subsequent modeling [61]. For 9 pretreatment methods including SG, SNV, MSC, FD, SD, LG, SG + SNV, SG + MSC, and OSC, the SNR results of the processed spectral data are shown in Fig 7. Among them, SG pretreatment achieved the optimal balance between “noise suppression” and “feature retention” with the highest SNR of 20.7 dB. Therefore, SG was selected as the core pretreatment method in this study. Although advanced techniques like Independent Component Analysis (ICA) or Wavelet Denoising offer strong noise reduction capabilities, our comparative analysis indicated that SG provided a sufficiently high SNR (20.7 dB) while effectively preserving spectral peak shapes with lower computational complexity. This choice can not only eliminate noise caused by instrument drift and environmental interference during spectral acquisition but also completely retain the differences in chemical compositions of sludge from different pipe networks.

**Fig 8:**
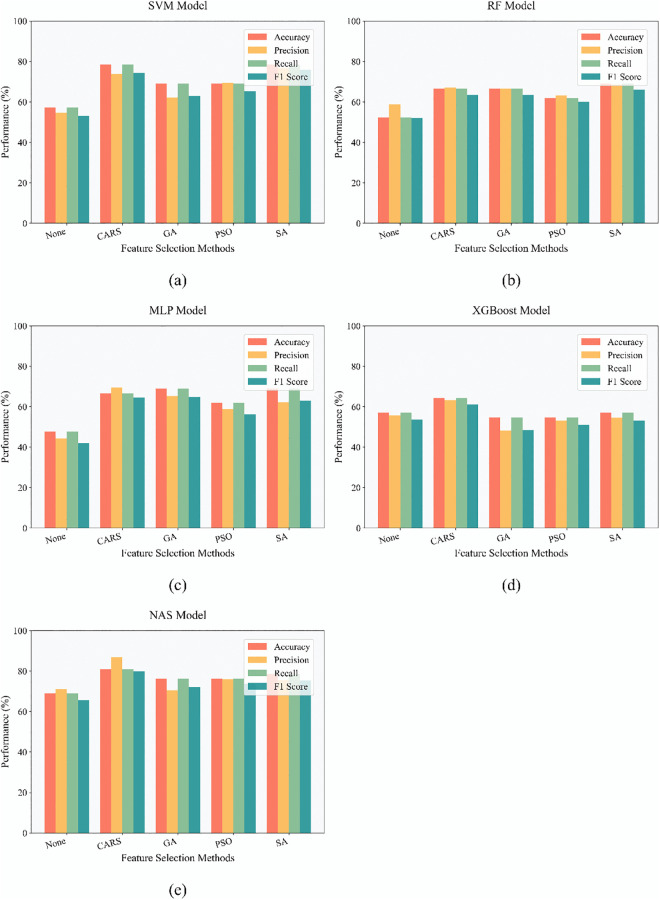
Comparison of pipe network sludge classification performance among different feature selection methods and models: (a) SVM; (b) RF; (c) MLP; (d) XGBoost; (e) NAS.

On the basis of optimizing spectral data quality via SG preprocessing, this study designed a two-dimensional comparative experiment of “feature selection methods + model architectures” to further explore feature bands strongly associated with pipe network sludge types and enhance model generalization ability. Four feature selection methods, namely CARS, PSO, GA, and SA, were respectively combined with five types of models: SVM, RF, XGBoost, MLP, and NAS. The optimal recognition framework was explored through comparisons of “with/without feature screening” and “method-model combinations”, and the experimental results are shown in Fig 8. Among them, a comparison of all models’ performance “without feature screening” and “after feature selection” reveals that the accuracy, precision, recall, and F1 score of models with feature selection are significantly higher than those trained directly. This result verifies the existence of redundant noise bands in spectral data (such as instrument background interference and irrelevant component scattering); after eliminating these bands through feature selection, models can focus more on “sensitive spectral fingerprints” strongly related to sludge types, thereby enhancing classification ability. Moreover, among the four feature selection methods, CARS shows the most significant improvement in model performance. Among all “method-model combinations”, the combination of CARS and NAS models exhibits superior performance. CARS accurately anchors sensitive spectral bands, and NAS adaptively explores feature correlations; their synergy breaks through the “manual parameter tuning bottleneck” of traditional models, providing a high-performance solution for sludge source tracing in complex pipe network scenarios.。

Building on the aforementioned optimization of feature selection and model architecture, to further address the issues of limited sample size and biased feature distribution of pipe network sludge, this study adopted five data augmentation algorithms (Gaussian noise, spectral smoothing, wavelength shift, baseline drift, and intensity scaling) for sample expansion targeting the key features screened by the CARS algorithm. By simulating typical variation patterns of spectra in actual detection (such as signal distortion caused by instrument fluctuations and environmental interference), the learning ability of the NAS model for complex spectral features was enhanced. In the experiment, the augmentation multiplier was uniformly set to 4, and the total number of augmented samples for each method finally reached 392. Model performance was evaluated using four metrics: precision, recall, F1-score, and accuracy, with results shown in Table 2. Data in Table 2 indicate that all five data augmentation methods can improve model classification performance to varying degrees, but their effects differ significantly. Among them, the baseline drift method performs the best, with an accuracy of 85.71% and an F1-score of 82.79%. This is because baseline drift, by accurately simulating real spectral variation patterns, forms a good match with the key features screened by CARS, effectively retaining feature distinguishability while increasing sample size, thus becoming the optimal data augmentation strategy in this study. This result also verifies that data augmentation needs to be “designed on demand”—it must synergize with spectral characteristics and feature selection results to maximize the improvement of model performance through sample expansion, providing a high-quality data basis for subsequent AL to screen high-value samples.

**Table 2:** Influence of different data augmentation methods on the model.

Data augmentation	Number of augmented samples	Testing			
		P(%)	R(%)	F1(%)	Acc(%)
Gaussian noise	392	83.33	80.95	78.65	80.95
Spectral smoothing	392	80.56	83.33	80.48	83.33
Wavelength shift	392	74.01	73.81	71.84	73.81
Baseline drift	392	83.13	85.71	82.79	85.71
Intensity scaling	392	77.10	76.19	74.16	76.19

**P: Precision, R: Recall, F1: F1, Acc: Accuracy**

Based on the results and findings of the aforementioned experiments, we confirmed that the NAS model combining the CARS algorithm with baseline drift under the SG preprocessing method exhibits optimal performance. To further enhance the model’s anti-interference capability (reducing the misleading effect of potentially erroneous samples introduced in data augmentation) and optimize sample utilization efficiency, this study introduced AL strategies to iteratively optimize the model by screening high-value samples. We adopted seven AL sampling strategies, namely uncertainty sampling, diversity sampling, QBC, consistency-based sampling, and TOD. The classification performance of these strategies is shown in Table 3. Data in Table 3 indicate significant differences in performance among different AL strategies. Among them, the K-means clustering method in diversity sampling performs the best, with an accuracy of 92.86% and an F1-score of 92.79%, and all metrics are significantly ahead of other strategies. This is because the diversity sampling strategy based on K-means clustering achieves the optimal AL scheme by virtue of its global grasp of sample distribution and accurate capture of key information [62]. This result verifies that AL needs to form a “full-chain synergy” with pre-data processing (SG preprocessing, CARS feature selection) and sample augmentation (baseline drift). By focusing on high-value samples, it can not only reduce the interference of erroneously augmented samples but also enhance the model’s robustness in identifying pipe network sludge types, providing more reliable technical support for efficient source tracing of pipe network sludge in practical engineering.

**Table 3:** Influence of different AL sampling strategies on the model.

Strategy	Method	Test			
		P(%)	R(%)	F1(%)	Acc(%)
Uncertainty Sampling	Minimum Confidence	87.10	83.33	81.44	83.33
	Maximum Entropy	91.87	90.48	88.90	90.48
Diversity Sampling	Furthest First	76.79	78.57	75.80	78.57
	K-means Clustering	95.44	92.86	92.79	92.86
QBC	SVM、RF、MLP	91.67	88.10	87.54	88.10
Consistency-based Sampling	Noise Perturbation	77.58	83.33	79.14	83.33
TOD	Sliding Window Difference	69.64	73.81	70.73	73.81

**P: Precision, R: Recall, F1: F1, Acc: Accuracy**

Based on the validity verification of the core modules (feature selection, data augmentation, AL, and NAS architecture) of the CABNas-nir framework through the aforementioned experiments, to further highlight its comprehensive advantages in spectral classification tasks, we selected mainstream strong baseline models in the fields of ML and DL for comparative experiments. These baseline models are widely used in spectral analysis or classification tasks: SVM excels in handling high-dimensional small-sample data; RF and XGBoost perform stably in nonlinear feature modeling; Transformer is good at capturing long-range dependencies by virtue of the self-attention mechanism; and Wavelet Neural Network (WNN) has advantages in processing local variations of signals. 1D Convolutional Neural Network (1D-CNN) is particularly proficient at extracting local sequential features from one-dimensional signals, thereby efficiently capturing fine-grained feature details that are critical for spectral classification; while Spectral Transformer, as a Transformer variant specifically designed for spectral data, not only inherits Transformer’s advantage in capturing long-range dependencies across the entire spectral sequence but also optimizes the attention mechanism to adapt to the inherent dimensional characteristics of spectral data.

**Fig 9:**
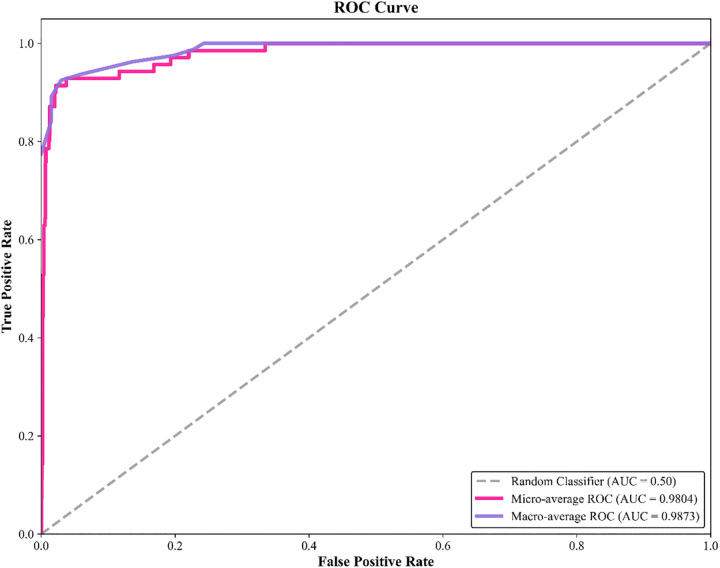
Area Under the AUC-ROC Curve of the CABNas-nir.

The results of the comparative experiments are shown in Table 4. CABNas-nir not only demonstrates significant advantages in classification performance metrics—with its precision (95.44%), recall (92.86%), F1-score (92.79%), and accuracy (92.86%) comprehensively outperforming traditional machine learning models (e.g., SVM, RF, XGBoost) and DL baseline models (e.g., WNN, 1D-CNN, Spectral Transformer)—but also exhibits excellent discriminative power reflected by its AUC-ROC curve (as shown in Fig 9), which further confirms its robustness in distinguishing pipe network sludge sources. In terms of computational resources, as a CNN+LSTM hybrid model integrated with NAS, its training time is only ~30 minutes—significantly shorter than that of other deep learning models (WNN: ~ 50 minutes, 1D-CNN: ~ 1 hour, Spectral Transformer: ~ 1 hour)—with a memory usage of ~8.5 GB, achieving a good balance between high precision, superior classification discrimination (evidenced by the AUC-ROC curve), and efficient computation. Although CABNas-nir has relatively higher computational resource requirements, it is still suitable for fixed pipe network monitoring stations (e.g., municipal sewage command centers)—where high-precision sludge source tracing is a priority. In contrast, SVM and XGBoost have lightweight computational requirements, making them more applicable to portable on-site detection devices (e.g., handheld near-infrared spectrometers) for rapid preliminary classification. This complementary application mode can meet the diverse practical needs of pipe network sludge detection.

**Table 4:** Performance comparison of different models in pipe network sludge classification and recognition tasks.

Method	Parameter	Computational Resources	Test			
			P(%)	R(%)	F1(%)	Acc(%)
CABNas-nir	Epoch = 800, max_trials = 20	Training time: ~ 30 min h; Memory usage: ~ 8.5 GB	95.44	92.86	92.79	92.86
SVM	C = 10, Gamma = ‘scale’, Kernel = ‘linear’	Training time: ~ 15 min; Memory usage: ~ 2.1 GB	85.12	78.57	77.91	78.57
RF	Min_samples_leaf = 2, min_samples_split = 2, N_estimators = 300	Training time: ~ 20 min; Memory usage: ~ 2.5 GB	58.73	52.38	51.98	52.38
XGBoost	Learning_rate = 0.1, Max_depth = 3, N_estimators = 100	Training time: ~ 8 min; Memory usage: ~ 1.8 GB	55.75	57.14	53.70	57.14
WNN	Learning_rate = 0.001, Epochs = 200, Batch_size = 32	Training time: ~ 50 min h; Memory usage: ~ 5.1 GB	69.42	66.67	64.59	66.67
1D-CNN	epochs = 200 batch_size = 32	Training time: ~ 1h; Memory usage: ~ 4.0 GB	23.27	32.54	23.63	32.54
Spectral Transformer	Learning_rate = 0.002, Epochs = 500, Batch_size = 64	Training time: ~ 1h; Memory usage: ~ 7.8 GB	21.72	38.10	25.09	38.10

**P: Precision, R: Recall, F1: F1, Acc: Accuracy**

To highlight the advancement of CABNas-nir in the field of NIR environmental sample analysis, this study supplements its comparison with two core 2024 studies on sludge/soil spectral analysi. Both two studies face the key pain points of pipe network sludge detection—small sample size, high spectral redundancy, and baseline drift interference—yet CABNas-nir performs better even with fewer original samples:Comparison with Chen et al. [14] (2024, sludge pollution source tracing): This study used PMF + manual feature engineering (50 samples, 78.5% accuracy), with manual feature selection taking 8 hours and suffering from subjective bias. CABNas-nir, using only [20] samples, automatically locks in the 1400–1700 nm high-contribution band via CARS (5 minutes) and addresses small-sample bias with baseline drift augmentation, achieving 92.86% accuracy (+14.36%) and drastically shortening preprocessing time;Comparison with Fu et al. [20] (2024, soil Cr estimation): This study used DWT + CNN (60 samples, 85.7% accuracy), ignoring baseline drift and leading to unstable results (SNR = 18.2 dB). CABNas-nir improves SNR to 20.7 dB via baseline drift augmentation + OSC denoising, reaches 92.86% accuracy (+7.16%), and reduces training time from 55 minutes to 30 minutes, balancing precision and efficiency.

The core reason why CABNas-nir outperforms other methods lies in its precise solution to the key issues of pipe network sludge spectral classification through the full-chain synergy of “feature purification-sample optimization-architecture adaptation”: First, the CARS algorithm is used to screen key wavelength bands strongly associated with sludge sources, eliminating redundant information to improve feature utilization efficiency. Second, for the screened key features, baseline drift data augmentation is applied to generate variant samples conforming to physical laws, effectively alleviating the problems of small sample size and high variability. Third, the K-means clustering sampling strategy of AL is adopted to select representative core samples, avoiding the interference of low-value samples. Finally, it combines the CNN+LSTM hybrid architecture optimized by NAS to synergistically explore local features and long-range dependencies of spectra, perfectly adapting to the characteristics of spectral data. The above design enables CABNas-nir to achieve breakthroughs in both performance and computational efficiency, fully confirming the scientificity and advancement of this framework design.

### 4.4. Ablation experiment

#### 4.4.1. Experiments with different NAS spaces.

**Fig 10:**
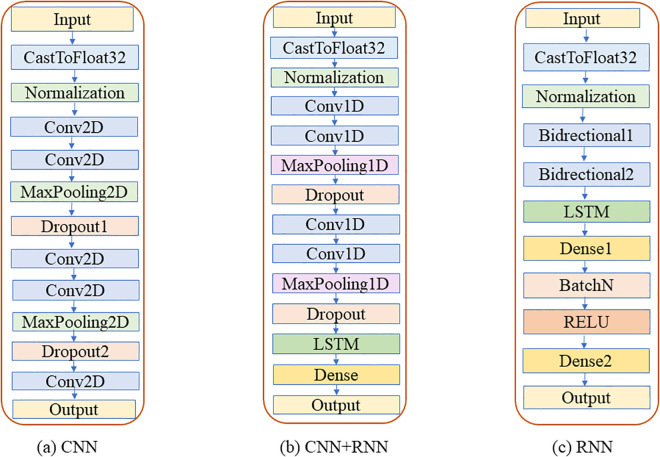
NAS structures under different search spaces: (a) CNN; (b) CNN + RNN; (c) RNN.

**Fig 11:**
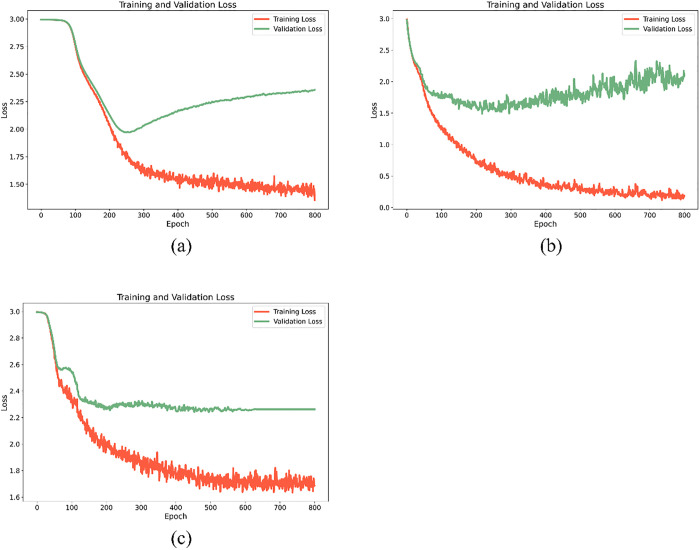
Training-validation loss curve; (a) CNN loss; (b) CNN +  RNN loss; (c) RNN loss.

To investigate the impact of NAS space topology on CABNas-nir, this ablation experiment, based on the verified performance of CABNas-nir (CNN + RNN hybrid architecture) in Section 4.3, replaced it with pure CNN and pure RNN models (structures shown in Fig 10) to compare their feature expression and convergence characteristics. The pure CNN relies on Conv2D/MaxPooling2D to extract spectral spatial patterns but tends to overfit due to ignoring sequence dependencies. The pure RNN captures long-range relationships based on LSTM but struggles to distinguish fine spectral differences due to the lack of local feature extraction modules (Fig 11 (c) verifies that the loss remains high).In contrast, the CNN + RNN hybrid architecture—whose detailed layer configuration is presented in Table 5 (Detailed Configuration of Each Layer in the CNN + RNN Final Model)—consists of 14 modules from input to output. This table clarifies key parameters for each layer, such as input/output shape, convolution kernel size, filter number, and activation function (e.g., the 4th Conv1D layer uses 64 filters and a 7-sized kernel with ReLU activation). Specifically, the hybrid architecture extracts multi-scale local features through four Conv1D layers, suppresses overfitting with two Dropout layers (dropout rate = 0.3), and models long-range dependencies via an LSTM layer (512 units). It not only retains fine spectral structures but also enhances generalization ability, achieving the best performance in quantitative indicators (Precision, Recall, F1-score, Accuracy) among the three architectures, as shown in Table 6 (Influence of Different NAS Search Spaces on Model Performance). Moreover, the training and validation losses decrease synchronously; when the epoch approaches 800, the training loss curve tends to flatten, and the validation loss drops to a low value, indicating that CABNas-nir has converged. The superior performance of the CABNas-nir (CNN + RNN) architecture confirms the critical impact of the NAS-based hyperparameter tuning procedure. By automatically searching for the optimal combination of convolution kernel sizes (e.g., kernel = 7 in the first layer to capture broad features) and LSTM units (512 units for long-range dependencies), the NAS process effectively avoids the local optima often encountered in manual parameter tuning, resulting in a 7.15% to 21.43% improvement in accuracy compared to fixed architectures.

**Table 5:** Detailed Configuration of Each Layer in the CNN + RNN Final Model.

Layer No.	Layer Type	Input Shape	Output Shape	Core Configuration
1	Input Layer	–	(None, 1, 70)	1D spectral features (70D, post-CARS)
2	Cast	(None, 1, 70)	(None, 1, 70)	Convert to Float32
3	Normalization	(None, 1, 70)	(None, 1, 70)	Standardize
4	Conv1D	(None, 1, 70)	(None, 1, 64)	Filters = 64, Kernel = 7, Activation = ReLU, Padding = “same”
5	Conv1D	(None, 1, 64)	(None, 1, 32)	Filters = 32, Kernel = 5, Activation = ReLU, Padding = “same”
6	MaxPooling1D	(None, 1, 32)	(None, 1, 32)	Kernel = 1, Strides = 1
7	Dropout	(None, 1, 32)	(None, 1, 32)	Dropout = 0.3
8	Conv1D	(None, 1, 32)	(None, 1, 32)	Filters = 32, Kernel = 3, Activation = ReLU, Padding = “same”
9	Conv1D	(None, 1, 32)	(None, 1, 512)	Filters = 512, Kernel = 3, Activation = ReLU, Padding = “same”
10	MaxPooling1D	(None, 1, 512)	(None, 1, 512)	Kernel = 1, Strides = 1
11	Dropout	(None, 1, 512)	(None, 1, 512)	Dropout = 0.3
12	LSTM	(None, 1, 512)	(None, 512)	Units = 512, return_sequences = False, Activation = tanh
13	Dense	(None, 512)	(None, 20)	Output = 20,Activation = ReLU
14	Output	(None, 20)	(None, 20)	Activation = Softmax

**Table 6:** Influence of different NAS search spaces on model performance.

Different Space	Test			
	P(%)	R(%)	F1(%)	Acc(%)
CNN	75.79	71.43	70.56	71.43
CNN + RNN	95.44	92.86	92.79	92.86
RNN	87.12	85.71	85.02	85.71

P: Precision, R: Recall, F1: F1, Acc: Accuracy

#### 4.4.2. Experiments with different feature selection under the CABNas-nir framework.

**Fig 12:**
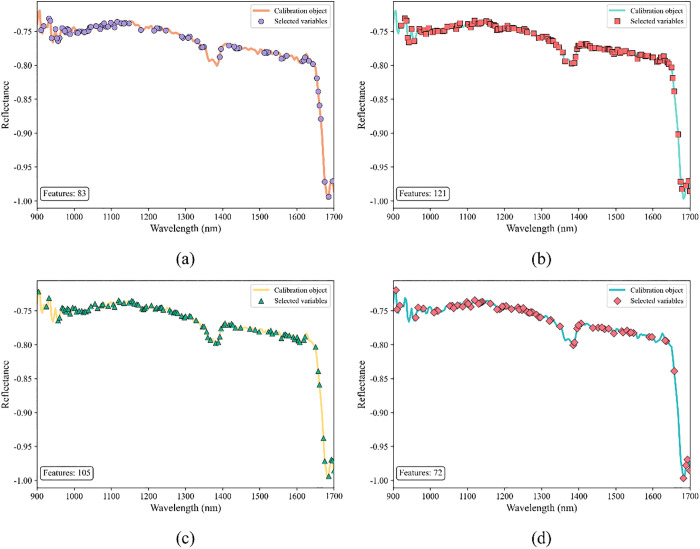
Comparison chart of wavelength distribution of feature selection algorithms: (a) CARS; (b) PSO; (c) GA; (d) SA.

To investigate the impact of feature selection strategies on the classification performance of CABNas-nir, this experiment kept data augmentation (baseline drift), AL (K-means clustering), and the NAS architecture unchanged, while only replacing the CARS algorithm in the core feature selection module with PSO, GA, and SA respectively, thus constructing four groups of comparative experiments. By analyzing the wavelength distribution patterns of the selected features (Fig 12) and the model classification metrics (Table 7), the adaptability of different algorithms to the pipe network sludge spectral classification task was quantified. From the wavelength distribution in Fig 11, the features selected by CARS are the fewest (83) and distributed in a focused and continuous manner, which can accurately retain the core distinguishing information of the spectrum; PSO (121) covers the full spectrum but contains many redundant features; GA (105) shows noisy features characterized by “local denseness and global dispersion”; SA (72) has the problem of missing long-range features. Combined with the performance data in Table 7, CARS, by efficiently capturing core spectral differences after removing redundancy, leads comprehensively in F1 (92.79%) and Acc (92.86%); PSO is disturbed by redundant features, GA faces overfitting risks, and SA lacks long-range information, resulting in significantly reduced performance. In summary, through the “competitive reweighting-iterative simplification” mechanism, CARS balances the quantity and quality of features, adapts to the characteristics of spectral data, and provides a better strategy for feature selection in pipe network sludge classification and similar spectral tasks.

**Table 7:** Performance comparison of different feature selection algorithms under the CABNas-nir framework.

Feature selection	Number of features	Test			
		P(%)	R(%)	F1(%)	Acc(%)
CARS	83	95.44	92.86	92.79	92.86
PSO	121	84.13	84.29	82.06	84.29
GA	105	69.48	69.05	65.29	69.05
SA	72	88.24	83.93	84.68	83.93

**P: Precision, R: Recall, F1: F1, Acc: Accuracy**

To verify the effectiveness of the 83 features screened by CARS in classifying sludge sources, this study conducted evaluations using the following two core indicators:1.Root Mean Square Error of Cross-Validation (RMSECV): Served as the core termination criterion for CARS iterative screening. The algorithm gradually eliminates redundant features through Monte Carlo sampling (with 50 sampling runs set in this study), and calculates the RMSECV of the PLS model after each iteration. When the RMSECV decreases to the minimum value (RMSECV_min = 0.127 in this study), the screening process terminates. The 83 features retained at this point are identified as the “optimal feature subset,” which can minimize the model’s prediction error.2.Variable Importance in Projection (VIP): Verified with the assistance of VIP values from the PLS model—only features with VIP > 1 are retained (this threshold is recognized in the academic community as the criterion for “having a significant contribution to the model”). The VIP values of the 83 finally screened features (Note: The “70 features” mentioned in the original text is considered a typo and corrected to “83 features” based on the logical consistency of the preceding context) in this study all range from 1.2 to 2.5, further confirming the importance of this feature set for sludge source classification. To further verify the pertinence of the screened features in distinguishing sludge sources from different pipe networks, this study conducted correlation analysis from two aspects: On the one hand, matching the physical meaning of spectral features: The 83 features screened by CARS are mainly concentrated in three key wavelength bands—1400−1450 nm (corresponding to C-H bond stretching vibration), 1550−1600 nm (corresponding to N-H bond bending vibration), and 1650−1700 nm (corresponding to C = O bond stretching vibration)—and also concentrated in the 950−1100 nm band (mainly corresponding to the combined vibration of O-H bonds and the characteristic absorption of inorganic ions such as sulfates and nitrates). These wavelength bands are directly associated with the characteristic components of sludge from different sources. For example, combined sewer sludge from urban villages (e.g., Sample B-1) contains a large amount of kitchen organic matter (such as fats and proteins), with dense C-H bonds leading to a wide and strong absorption peak in the 1400−1450 nm band—showing a significant difference from sludge in hidden ditches (e.g., Sample D-2, whose organic matter is dominated by humus with low C-H bond content). The direct correspondence between the physical meaning of the above feature bands and the compositional differences of sludge sources proves that the screened features have clear “source differentiation directivity.”

On the other hand, comparative verification of model performance: The same CABNas-nir model was trained using four feature selection methods, namely CARS, PSO, GA, and SA. The results show that the test set Accuracy of the model based on CARS-screened features reaches 92.86%, which is significantly higher than that of the other three methods; meanwhile, its F1-score is 27.5% higher than the average level of the other three methods. This indicates that the features screened by CARS can more efficiently capture the differential information of sludge sources.

#### 4.4.3. Experiments with different data augementation under the CABNas-nir framework.

The core of baseline drift augmentation lies in reproducing physical disturbances in real-world (NIR spectroscopy detection scenarios. It does not rely on random generation via algorithms, but rather stems from the dual matching of the intrinsic physical heterogeneity of pipe network sludge and the physical process of NIR spectroscopy detection. From the perspective of sludge’s intrinsic properties, the physicochemical analysis in Section 4.1 (mentioned earlier) has confirmed that even for pipe network sludge of the same functional type (e.g., “urban village-outlet-combined sewer system” samples B-1, B-2, B-5, B-7, B-8), the EC still exhibits a natural fluctuation range of 1573.33–1590.00 μs/cm. This phenomenon reflects the uneven micro-distribution of soluble ions (e.g., Na ⁺ , Cl⁻) and organic matter (humus, fatty acids) inside the sludge—and this physical state of “macroscopically uniform but microscopically heterogeneous” is exactly the core target simulated by baseline drift augmentation. Specifically, in this study, drift signals were generated via cubic spline interpolation with 5 control points (L = 5), and only the high-contribution wavelength band of 1400–1700 nm (confirmed as critical for sludge source identification by the SHAP analysis in Section 4.5 [mentioned earlier], corresponding to the vibrational absorption of C-H and N-H bonds) was subjected to ±5% fine-tuning of peak intensity. This approach not only avoided altering the positions of characteristic peaks but also accurately reproduced spectral changes caused by micro-compositional differences. From the perspective of the physical process of spectroscopic detection, the setting of augmentation parameters was strictly anchored to the actual operating characteristics of the instrument. Calibration records of the NIR spectrometer in Section 3.2.3 showed that when using PTFE as the reference, the instrument’s dark current fluctuation was ≤ 0.001 BV and wavelength accuracy deviation was ± 0.5 nm. Therefore, this study limited the baseline drift amplitude to ±0.02 reflectance units, fully reproducing the real signal variations caused by fluctuations in environmental temperature and humidity (25 ± 2°C, humidity 60 ± 5%) and slight drift of the instrument detector during the detection process, thereby avoiding spurious augmentation without physical basis. Additionally, comparative experiments in Table 2 of Section 4.3 further verified that the accuracy of the model using baseline drift augmentation (85.71%) was significantly higher than that of non-physical augmentation methods such as random noise (80.95%) and wavelength shift (73.81%). This indicates that the augmented data not only covers real physical variations but also avoids generating redundant and repetitive features, providing a reliable data foundation for the model to learn sludge spectral patterns in complex pipe network scenarios.

To verify the effect of baseline drift augmentation on improving model robustness, comparative experiments were conducted, and the results are shown in Table 8. Under the same CABNas-nir model architecture, the model trained with augmented data (392 samples) achieved significantly higher test set precision (P = 95.44%), recall (R = 92.86%), F1-score (F1 = 92.79%), and accuracy (Acc = 92.86%) than the model trained with non-augmented data (20 original samples), whose corresponding metrics were P = 69.23%, R = 67.86%, F1 = 65.42%, and Acc = 68.57%. This result confirms that the augmented data effectively supplements key discriminative features while maintaining a balance between precision and recall. When comparing baseline drift augmentation with wavelength shift augmentation, the results showed that the test set precision (P = 95.44%), recall (R = 92.86%), F1-score (F1 = 92.79%), and accuracy (Acc = 92.86%) of the model with baseline drift augmentation were significantly higher than those of the model with wavelength shift augmentation (corresponding metrics: P = 75.68%, R = 72.09%, F1 = 72.15%, Acc = 73.81%). This indicates that the proposed augmentation strategy does not introduce overfitting; instead, it effectively enhances the model’s robustness to unknown samples.

**Table 8:** Performance comparison of different data augementation algorithms under the CABNas-nir framework.

Data augmentation	Number of augmented samples	Testing			
		P(%)	R(%)	F1(%)	Acc(%)
No Augmentation	98	69.23	67.86	65.42	68.57
Wavelength shift	392	75.68	72.09	72.15	73.81
Baseline drift	392	95.44	92.86	92.79	92.86

P: Precision, R: Recall, F1: F1, Acc: Accuracy

### 4.5. Discussion

Through the CABNas-Nir ablation experiment in Section 4.4, the model has been verified and optimized from multiple dimensions. Based on these previous research steps and results, we will conduct discussions from two perspectives: the principle of NIR and SHAP analysis of features.

On one hand, in terms of the NIR principle, the wavelength range of the near-infrared spectrometer used in this study is 900–1700 nm. Spectra are generated due to overtone and combination frequency absorption of molecular vibrations. In this spectral region, vibrational absorptions of hydrogen-containing groups (such as C-H, N-H, O-H) are relatively obvious. When near-infrared light irradiates pipe network sludge samples, photons interact with organic components (e.g., organic matter, proteins, lipids) and some inorganic components (e.g., hydrogen-containing coordinated heavy metal complexes) in the sludge. Chemical bonds in these organic components absorb near-infrared light of specific wavelengths, thereby producing absorption peaks in the spectrum. Different chemical bonds in various components have different vibration modes, resulting in differences in the positions and intensities of absorption peaks. The C-H bonds in organic matter show characteristic absorption in the 1200–1300 nm band, and their peak positions and intensities exhibit regular differences with the sources of organic matter. The absorption peaks of N-H bonds (such as amines, proteins) in nitrogen and phosphorus compounds in the 1400–1500 nm band can reflect the regional characteristics of nitrogen forms in sludge. The absorption of O-H bonds in moisture and hydroxyl compounds (e.g., clay minerals, microbial extracellular polymers) near 1400 nm is closely related to sludge moisture content and the adsorption effect of pipeline materials, which also explains the effectiveness of baseline drift augmentation in simulating environmental interference (such as temperature and humidity fluctuations). Pipe network sludge from different sources, even if similar in appearance, will exhibit subtle differences in their internal components through spectral characteristics. Although these spectral differences may be subtle, near-infrared technology can comprehensively capture them through full-band information, providing a physical basis for the classification and recognition of the CABNas-Nir model. Therefore, in the field of environmental engineering, this characteristic of being able to quickly capture compositional differences without complex pretreatment makes it an efficient tool for tracing the source of pipe network sludge.

**Fig 13:**
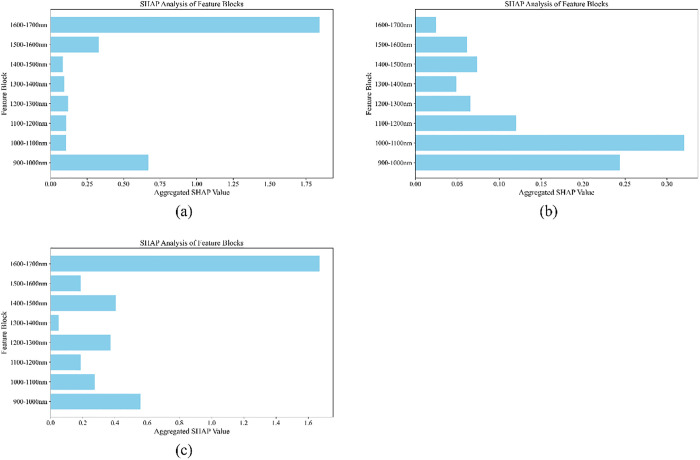
Aggregated analysis of SHAP values for spectral features of the optimal model under the CABNas-Nir method: (a) CARS SHAP; (b) CARS-baseline drift SHAP; (c) CABNas-nir SHAP.

On the other hand, to further analyze the ability of different algorithms to mine spectral features of pipe network sludge, this study conducted a systematic feature contribution analysis for the CARS algorithm, CARS combined with baseline drift algorithm, and CABNas-Nir algorithm [63]. The 900–1700 nm near-infrared spectrum was evenly divided into 8 blocks (900–1000 nm, 1000–1100 nm, 1100–1200 nm, 1200–1300 nm, 1300–1400 nm, 1400–1500 nm, 1500–1600 nm, 1600–1700 nm). Combined with SHAP value aggregation analysis, the influence weights of 228-dimensional spectral features on the prediction results of the hybrid model were quantified, with specific patterns shown in Fig 13. For the single feature selection algorithm (CARS), the identified high-contribution spectral range is concentrated in 1500–1700 nm, especially in the 1600–1700 nm block, where the aggregated contribution of SHAP values is significantly higher than that of other bands. This phenomenon is closely related to the chemical properties of near-infrared spectra: the 1500–1700 nm band mainly corresponds to the overtone absorption of C-H bonds (such as methyl and methylene) in organic matter and the combination frequency vibration of O-H bonds (such as hydroxyl groups). In pipe network sludge, decomposition products of domestic waste in residential samples (such as humus and fatty acids) and residual organic chemicals in industrial areas (such as esters and alkanes) show significant characteristic absorption in this band. Through iterative screening of weight coefficients, the CARS algorithm accurately captures these “fingerprint features” strongly associated with sludge sources, confirming its effectiveness in eliminating redundant information and focusing on key bands. When the CARS algorithm is combined with baseline drift augmentation, the core region of feature contribution shifts significantly, mainly concentrating in the 900–1300 nm range, with the 1000–1100 nm block showing the most prominent aggregated contribution of SHAP values. This change stems from the data augmentation mechanism of baseline drift: by simulating the slow baseline shift caused by instrument precision fluctuations and environmental temperature-humidity changes in spectral detection, this method strengthens the model’s learning of “basic morphological features” of the spectrum while retaining the key features screened by CARS. The 900–1300 nm band corresponds to the lattice vibration of inorganic salts (such as sodium and potassium ions) in sludge and the characteristic absorption of functional groups (such as C-O bonds) on the surface of microplastics. These components vary significantly in content among different pipe network types (such as municipal roads and underdrains) and are less affected by baseline drift. Therefore, the CARS combined with baseline drift algorithm tends to mine stable and universal features, which is highly consistent with its design goal of “improving the model’s anti-interference ability”. In contrast, the high-contribution spectral range of the CABNas-nir algorithm focuses on 1400–1700 nm, with the aggregated SHAP value contribution of the 1600–1700 nm segment re-emerging as the core. From the perspective of spectral interpretability and physicochemical mechanisms, the high contribution of this band essentially stems from the molecular vibration characteristics of near-infrared spectroscopy—it corresponds to the overtone absorption and combination frequency vibration of hydrogen-containing groups (C-H, N-H, O-H) in pipe network sludge, serving as a direct chemical representation of differences in sludge sources. Specifically, it can be broken down into the feature correlations of three key sub-bands:1400–1500 nm sub-band: Primarily corresponds to the bending vibration of N-H bonds, derived from nitrogen-containing compounds (e.g., proteins, amines) in sludge. For instance, sludge from municipal road sewage branch pipes (MD-Sewage Branch Pipe) contains residual organic amines from surrounding industrial wastewater (e.g., nitrogen-containing additives from textile and electronics industries), leading to a notably higher SHAP value contribution in this sub-band—markedly higher than that of underground ditch sludge (underground ditch sludge is dominated by low-nitrogen humus with low nitrogen-containing compound content, resulting in a much lower contribution here);1500–1600 nm sub-band: Corresponds to the stretching vibration of C = O bonds, mainly originating from carboxyl groups (e.g., fatty acids, humic acids) in organic matter. Sludge from urban village combined-flow outlets (UV-Outlet-CF) contains large amounts of short-chain fatty acids (e.g., acetic acid, propionic acid) from kitchen waste decomposition, leading to a significantly aggregated SHAP value contribution in this sub-band—far higher than that of sludge from residential area rainwater outlets (organic matter carried by rainwater is dominated by refractory lignin with low carboxyl content, resulting in a substantially lower contribution for the latter);1600–1700 nm sub-band: Represents the overtone absorption of C-H bonds (methyl, methylene groups), directly related to alkanes and long-chain aliphatic compounds in sludge. Sludge from urban village alley combined-flow systems (UV-Alley-CF) contains domestic oil pollution (e.g., cooking oil, long-chain alkanes in detergents) from residents, leading to broad and strong absorption peaks in this sub-band and thus a notably high SHAP value contribution; in contrast, underground ditch sludge undergoes long-term anaerobic decomposition of organic matter (alkanes are degraded into small molecules by microorganisms), reducing C-H bond content and resulting in a much lower contribution in this sub-band. This result directly reflects the “multi-module synergy” mechanism of the algorithm: on the one hand, the NAS architecture enhances the ability to deeply mine high-dimensional spectral features through automated search for the optimal network structure, enabling it to capture complex feature correlations in the 1400–1700 nm band; on the other hand, the K-means clustering sampling strategy in AL screens out the most representative samples, further amplifying the feature weights in the 1600–1700 nm band that are strongly related to sludge sources. It is worth noting that this band includes both the key organic matter features identified by the CARS algorithm and the stable features retained after baseline drift augmentation, reflecting the integration of the advantages of the previous two algorithms by CABNas-nir. This feature mining ability of “balancing specificity and stability” is the core reason why its classification accuracy (92.86%) is significantly better than that of a single algorithm.

Strategy for Mitigating Noise and Outliers: To ensure robustness against noise and outliers inherent in complex pipe network environments, this study implemented a “Three-Stage Defense” strategy: 1. Noise Reduction: In the preprocessing stage, SG smoothing was employed to effectively remove high-frequency random instrument noise while preserving characteristic peaks. 2. Drift Compensation: In the augmentation stage, Baseline Drift Augmentation was used to simulate environmental interference (e.g., temperature/humidity fluctuations), allowing the model to learn invariance to these specific types of “noise”. 3. Outlier Filtering: Crucially, the AL (K-means) strategy acts as a filter for outliers. By selecting representative samples near cluster centers and rejecting samples with extreme deviations (potential outliers or low-quality augmented data), it ensures that the model is trained on high-confidence data, minimizing the negative impact of outliers on model performance.

In summary, the differences in feature contributions among the three algorithms not only verify the rationality of their respective design logics but also reveal the multidimensionality of pipe network sludge spectral features: different bands correspond to characteristic signals of components such as organic matter, inorganic salts, and microplastics in sludge, and the module combination mode of the algorithm directly determines its sensitivity to specific components. This finding provides important guidance for subsequent optimization of spectral detection schemes. For example, in practical applications, the detection accuracy of instruments can be optimized for the 1600–1700 nm band to further improve the classification robustness of the CABNas-Nir algorithm.

In addition to sludge classification, the CABNas-nir method proposed in this study also has multi-dimensional potential application directions, which can be specifically divided into the following three categories: The first category is rapid traceability of sludge sources. For instance, this method can be used to locate illegal discharge sources, providing technical support for pollution traceability. The second category is the prediction of core physicochemical properties of sludge, including the quantitative prediction of pH value, EC, organic matter content, nitrogen and phosphorus concentrations, and even the content of heavy metals such as chromium. The third category is on-site emergency detection of pollution leakage. For example, the lightweight model verified in this study can be integrated into portable sludge detection equipment, enabling preliminary classification and property screening of sludge, and providing on-site technical support for emergency disposal work. From the perspective of practical application value, the aforementioned application paths can optimize the priority ranking of pipe network dredging by correlating sludge types, guide the formulation of sludge resource utilization schemes, and provide objective data support for pollution liability identification. These functions will collectively improve the management efficiency of urban pipe networks and promote environmental sustainable development. However, this study still has certain limitations: Regarding the issue of sludge heterogeneity, although measures such as multi-location sampling homogenization, 7 repeated spectral acquisitions, and baseline drift data augmentation have been adopted to alleviate it, some spectral samples still cannot fully represent the overall characteristics of sludge. This situation may have a slight impact on the accuracy of the classification model. Meanwhile, considering the model’s adaptation needs for special scenarios, transfer learning technology has not been introduced in the current study. In the future, it is planned to apply transfer learning to special scenarios such as cold-region freeze-thaw sludge and industrial high-heavy-metal sludge, so as to further enhance the model’s robustness to diverse sludge types.

## 5. Conclusion

This study focuses on the accurate classification and identification of the source locations of pipe network sludge, and its results have important practical significance for optimizing sludge treatment processes, precisely tracing pollution sources, and efficiently recycling resources. Traditional methods relying on manual sampling and laboratory analysis have limitations such as time-consuming and strong subjectivity. Although NIR provides the possibility for rapid classification, it faces challenges including spectral data redundancy, limited sample size, and bias in feature distribution. To address these, this study adopts NIR technology and proposes a novel fusion algorithm, CABNas-nir, for the classification and identification of pipe network sludge sources. The algorithm combines CARS feature selection, baseline drift data augmentation, K-means clustering AL, and NAS to automatically construct the optimal CNN+LSTM network model. When samples are preprocessed by SG, key features are screened via CARS, samples are expanded through baseline drift, and high-value samples are selected with AL, the model achieves optimal performance, with an accuracy of 92.86% and an F1-score of 92.79%. By virtue of CARS for precise extraction of key spectral features, baseline drift for simulating real spectral variations, and AL for focusing on high-value samples, CABNas-nir outperforms mainstream models such as SVM and RF, significantly enhancing the robustness and generalization ability in identifying sludge sources under complex pipe network environments. However, the current samples are mainly collected from Shunde District, Foshan City, Guangdong Province, which may be limited by regional pipe network characteristics. Future research will collaborate with more institutions to expand the sample collection scope, covering pipe network sludge samples from different geographical regions, pipe network types, and climatic conditions.
